# Modeling dopaminergic and other processes involved in learning from reward prediction error: contributions from an individual differences perspective

**DOI:** 10.3389/fnhum.2014.00740

**Published:** 2014-09-30

**Authors:** Alan D. Pickering, Francesca Pesola

**Affiliations:** ^1^Department of Psychology, Goldsmiths, University of LondonLondon, UK; ^2^Section for Recovery, Health Service and Population Research Department, Institute of Psychiatry, King's College, University of LondonLondon, UK

**Keywords:** dopamine, extraversion, reward prediction error, feedback related negativity (FRN), computational modeling of human behavior, reinforcement learning models

## Abstract

Phasic firing changes of midbrain dopamine neurons have been widely characterized as reflecting a reward prediction error (RPE). Major personality traits (e.g., extraversion) have been linked to inter-individual variations in dopaminergic neurotransmission. Consistent with these two claims, recent research (Smillie et al., 2011; Cooper et al., 2014) found that extraverts exhibited larger RPEs than introverts, as reflected in feedback related negativity (FRN) effects in EEG recordings. Using an established, biologically-localized RPE computational model, we successfully simulated dopaminergic cell firing changes which are thought to modulate the FRN. We introduced simulated individual differences into the model: parameters were systematically varied, with stable values for each simulated individual. We explored whether a model parameter might be responsible for the observed covariance between extraversion and the FRN changes in real data, and argued that a parameter is a plausible source of such covariance if parameter variance, across simulated individuals, correlated almost perfectly with the size of the simulated dopaminergic FRN modulation, and created as much variance as possible in this simulated output. Several model parameters met these criteria, while others did not. In particular, variations in the strength of connections carrying excitatory reward drive inputs to midbrain dopaminergic cells were considered plausible candidates, along with variations in a parameter which scales the effects of dopamine cell firing bursts on synaptic modification in ventral striatum. We suggest possible neurotransmitter mechanisms underpinning these model parameters. Finally, the limitations and possible extensions of our general approach are discussed.

## Introduction

For researchers investigating the psychobiological basis of personality, the basic hypothesis they interrogate is as follows: psychobiological process X is specifically associated with personality trait A. There is an implication that variance in process X *causes* a proportion of the variance in the trait, but the evidence obtained is unlikely to clarify the causal direction of the association (unless a genetic or genomic design is employed). For example, trait A might be extraversion (the focus of the current article) and we will consider possible candidates for process X below.

This article contends that a major issue for psychobiological personality research is the under-specification of the psychological processes, with serious implications for the proper construction of tasks that are designed to measure them. We have made this point previously a number of times in the last two decades (e.g., Pickering et al., 1997), and have thus proposed that formal modeling of the tasks involved may be necessary in order to derive stronger evidence (e.g., Pickering, 2008). In this paper, we will show how having a formal and biologically-mapped model can begin to clarify our attempts to measure specific psychological processes.

## A general modeling framework for differential computational neuroscience (DCN)

To understand how we try to investigate individual differences using computational methods, it is important first to lay out, in some detail, the 6-step approach which we are adopting. For convenience we refer to it as the differential computational neuroscience (DCN) framework. The six steps of the approach we follow are summarized in Table 1. This paper presents in detail how we have prosecuted steps 1–4 in relation to a specific laboratory task that we have used to study extraversion. Steps 5 and 6 are briefly considered in the discussion.

**Table 1:** **The six stages of the differential cognitive neuroscience framework**.

1. Identify possible endophenotypes for the trait of interest (here, extraversion).
2. Gather evidence to test whether the endophenotypes and the trait are associated/correlated.
3. Use cognitive neuroscience/computational models, and particularly those which have potentially biologically identifiable parameters, to simulate the target endophenotype (or processes contributing to endophenotypic variance).
4. Carry out the “individual differences” simulations of the endophenotype (or endophenotypic processes). This is done by allowing several “psychobiologically interpretable” candidate parameters to vary in the model, one by one. This sensitivity analysis is carried out to identify the best (i.e., most plausible) of the candidate parameters, using psychometric criteria.
5. Test the most plausible, specific model parameters for their ability to simulate variation in other distinct, but conceptually related, endophenotypes.
6. Explore whether, and in what contexts, the proposed parameter variation can simulate aspects of extraverted behavior in “toy” models.

### Step 1: identify possible endophenotypes for extraversion

As we will show below, we are going to use the DCN approach to explore whether extraversion might in part be driven by variance in a specific psychobiological process. We will try to relate that process to a specific parameter in a chosen bio-computational model. It might therefore appear most logical to use the model to try to simulate extraversion itself. However, a personality trait such as extraversion is typically extremely complex, being influenced by a raft of diverse causal influences, and usually being measured by a multi-item, self-report questionnaire. These properties make it intrinsically awkward to create a computational model which simulates a personality trait directly, although some interesting attempts to do so have been proposed (Read et al., 2010). Therefore, we instead focus upon simulating a so-called *endophenotype* (Gottesman and Gould, 2003).

The classic reason for using an endophenotype in psychiatric genetics is that the endophenotype “represents simpler clues to the genetic underpinnings than the disease syndrome itself (Gottesman and Gould, 2003, p. 636).” In the field of personality traits one might propose, in the same way, that the endophenotype gives us simpler clues to the causal bases (genetic and other) of the trait of interest. This means that the endophenotype is influenced by fewer underlying causal variables than the trait. Therefore, by studying endophenotypes, researchers have greater power to detect the relationships between specific causal variables/parameters and the endophenotype than they would if they were exploring the relationships between specific causes and the phenotype itself ^1^.

**Figure 1:**
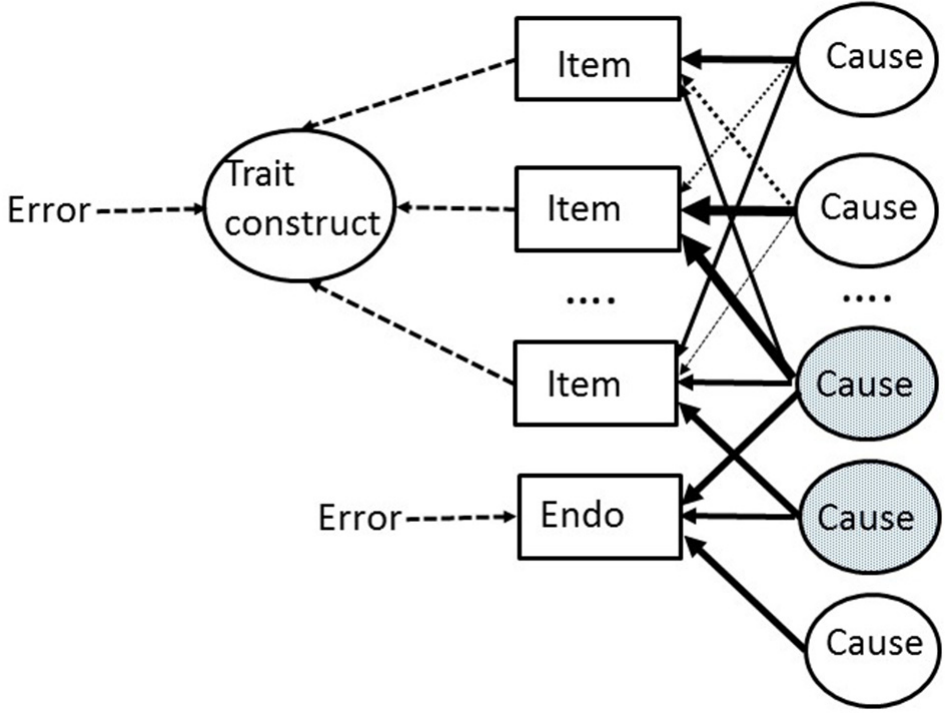
**An outline model of the relationship between underlying causes (of all kinds) and their action on: (a) self-report items used to measure a personality trait; and (b) an endophenotype (“Endo”)**. There are many more causes and items than are depicted. The relative weighting of the arrows from causes to measured entities (shown in rectangles) represent differing strength of causal influence. The endophenotype is affected by a smaller number of causes than the total number of causes which affect the phenotype (the trait) via its constituent trait items. Causes which affect both the endophenotype and some trait items are shaded. Error denotes measurement error. See text for more details.

Figure 1 makes the above arguments about the psychometrics of endophenotypes completely explicit. In Figure 1, a set of items are shown which are part of a so-called “formative model” in which the resulting personality trait construct is a convenient mathematical representation of the items (measures) which are used to form it (by using standard factor analytic methods). The items (usually considerably more than the 3 actually depicted) are all measured with error, which can be aggregated into a single error term (or disturbance term) acting on the whole construct. A large number of underlying causes are assumed. These might, for example, reflect: genetic variations; sensitivities of specific receptors in parts of the brain; levels of neurotransmitters available within specific brain pathways; structural variations in brain anatomy; the long-term consequences of specific life events and experiences; learned representations of events and outcomes in the world etc. etc. Once again, only a small number of the actual causes are depicted, and the figure does not imply that there are the same number of causes as items. The causes are shown as all independent of one another. The connections between causes and measures (items) are depicted in Figure 1 via a matrix of highly varying weights or loadings. Similarly, a single endophenotype is shown. This is depicted as having a partially overlapping set of underlying causes with some of the trait items (the shared causes are shaded in the figure). These shared causes are responsible for the observable correlation between the endophenotype and trait construct. Because these shared causes represent a (small) subset of the causes contributing variance to the trait construct, the observable correlation between the endophenotype and trait may often be quite weak and thus hard to detect reliably.

The specific choice of endophenotype has little bearing on the steps followed in the DCN modeling approach; the endophenotype that is to be modeled just has to be something which our to-be-chosen computational neuroscience model can simulate, and which is known to be associated with the actual phenotype of interest (in this case extraversion).

What specific endophenotypes might we propose as useful in the study of extraversion? The choice of endophenotype made below reflects two broad proposals about the causal basis of extraversion. The first proposal, first made in a seminal paper by Gray (1970), is that varying levels of extraversion reflect varying “responsiveness to reward cues.” In particular, much emphasis has been put on “learning of behaviors in rewarding contexts” (for an updated perspective and review, see Smillie et al., 2006). The second proposal, is the linking of extraversion to variation across individuals in aspects of dopaminergic neurotransmission (Depue and Collins, 1999; Pickering and Gray, 1999; Wacker et al., 2006; DeYoung, 2010, 2013; Depue and Fu, 2013). This proposed association between extraversion and processes linked to dopaminergic neurotransmission is the focus of this Frontiers research topic. The association is consistent with reported significant relationships between DA-related genes and measured extraversion (e.g., Smillie et al., 2010), or by psychopharmacological studies showing that extraversion modulates the effects of dopaminergic drugs (e.g., Wacker et al., 2006). However, DeYoung's (2010) paper presents evidence that several traits, other than extraversion, have been linked to variations in dopaminergic neurotransmission, thereby undermining the specificity of the extraversion-dopamine link. Some studies have tried to address this: Depue and Fu (2013) found links between extraversion and conditioned contextual activation of separate measures of affective, cognitive, and motor behavior; they induced the contextual conditioning of drug effects by using the dopamine-releasing drug methylphenidate. Their design used extreme groups (upper and lower deciles) of extraversion, but they attempted to control for the influence of other major traits (neuroticism, impulsivity) by also selecting participants who were in the middle six deciles on these other traits.

Based on these two long-standing proposals, we therefore decided to investigate an endophenotype which: (a) is linked to learning in a rewarding context; and (b) has a partially dopaminergic basis. To make a specific choice, one can draw upon the huge literature describing how dopaminergic neurotransmission is affected by encountering rewarding stimuli and learned cues that predict such rewards. A major element of this literature has come from studies addressing the function of midbrain dopamine (DA) neurons located in regions such as the ventral tegmental area and substantia nigra (Berridge and Robinson, 1998; Schultz, 1998, 2010; Bromberg-Martin et al., 2010; Glimcher, 2011). A major conceptualization has been that firing of these DA neurons does not reflect rewarding events *per se*. Instead, phasic increases and decreases in their firing is widely held to reflect a reward prediction error (RPE): that is, a discrepancy between the expected reward and the actual reward experienced. If the predicted reward is smaller than that received this generates a positive RPE; if the predicted reward is smaller than that received, then this leads to a negative RPE. This account has especially excited computational modelers (e.g., Glimcher, 2011), as RPE signals are a central component of many types of reinforcement learning models; in such models an RPE signal is often used to control learning. Thus, if a response is associated with a positive RPE, then the system learns that such a response should be repeated more frequently in that context (as it gains more reward than anticipated); if a response generates a negative RPE then that response should be executed less often in that context (as it generates less reward than expected). When the RPE is zero the response reliably produces its predicted reward and learning is complete.

**Figure 2:**
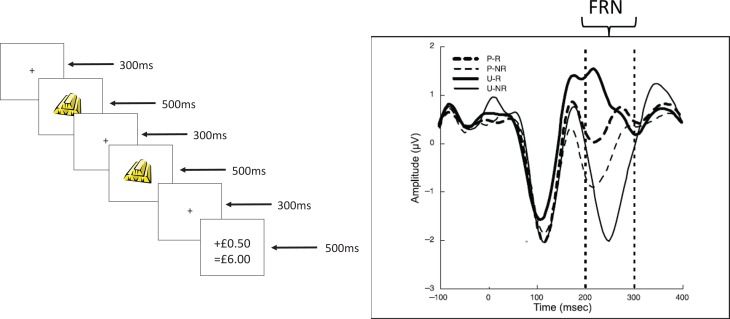
**(Redrawn from published originals)**. **Upper panel**. A typical trial from the Potts associative reward learning task. The times shown on the right hand side are the durations of each screen in ms. The particular task shown is an expected reward trial (see text for details). The final screen for each trial in the real task clearly indicated the reward gained on that particular trial (either nothing or 50 UK pence in our versions of the task) and the cumulative winnings. In this figure, the amount won on this trial is schematically depicted as “+ £0.50” and the amount of cumulative winnings is schematically depicted as “= £6.00.” **Lower panel**. A typical event related waveform from the task recorded with time=0 being the onset of stimulus 2 (S2). The feedback related negativity (FRN) is recorded in the period between the dotted lines (and is referenced to a suitable baseline period, prior to S2 onset). Waveforms for each of the 4 different trial types (P-R, predicted reward; P-NR, predicted non-reward; U-R, unpredicted reward; U-NR, unpredicted non-reward) are shown.

Based on the foregoing it is possible to suggest that one or more of the processes causing variation in the size of RPEs, through variations in phasic changes in DA cell firing rates, might be potential causal factors contributing to extraversion. If so, then we might profitably use an endophenotype which reflects these processes. In this paper we will consider an endophenotype derived from EEG recordings which arguably both reflects RPE processes and also depends on the firing of midbrain DA cells. We focus on the so-called ***feedback related negativity*** ^2^ (FRN). The FRN is a widely-studied negative-going event-related potential which typically occurs 250–300 ms after negative feedback, over mid-central scalp sites. It is typically computed as a difference between the waveforms for trials in which there is negative feedback, relative to trials on which there is positive (or less negative) feedback (see Walsh and Anderson, 2012, for a comprehensive review). A fairly typical example of the FRN is illustrated in Figure 2 below.

A major interpretation of the FRN has been that it reflects a quantitative RPE. This idea has been the subject of intense discussion and investigation (see Walsh and Anderson, 2012; Hauser et al., 2014, for details). In their comprehensive review, Walsh and Anderson (2012) concluded that the FRN did reflect a quantitative RPE. They cited 32 studies which supported this conclusion against 7 that provided contradictory evidence (see their Table 1).

The FRN (and related waveforms) has also been argued to arise from generators in medial prefrontal cortical structures such as the anterior cingulate cortex (ACC; see Holroyd and Coles, 2002). When reviewing FRN source localization studies Walsh and Anderson (2012) found 10 studies which had localized the FRN to the anterior cingulate and, although alternative and additional source loci were identified in other studies, they concluded that the ACC was the most consistently identified source of the FRN. The ACC is densely innervated by ascending mesocortical dopaminergic projections, and so it has also been widely argued (since Holroyd and Coles' original paper) that RPE signals, arising from the dopaminergic midbrain cells, act to modulate the ACC-generated FRN to give patterns of EEG waveforms such as those shown in Figure 2 below. Below, we will implement a typical biocomputational model of learning from RPEs. We will use the model to simulate changes in midbrain DA cell firing during an associative reward learning task. This task reliably elicits an FRN that varies in size depending on the prediction of reward as conveyed by the stimuli presented on a particular trial. These (simulated) DA cell firing changes will be our critical psychobiological process contributing to the endophenotype of interest (the FRN waveform differences across the conditions of the task).

From the above, it is clearly important that we also consider the robustness of the DA-modulation account of the FRN, all the more so as this popular viewpoint has recently been the subject of intense scrutiny (see Ullsperger et al., 2014, for a recent authoritative analysis). Ullsperger et al's review posed three key questions that are highly relevant; they asked whether FRN and related phenomena: (a) are influenced by dopaminergic transmission; (b) whether and how the dopaminergic system might code negative RPEs; and (c) whether a change in the firing rate of the mesocortical dopaminergic neurons can result in the modulation of ACC generators of FRN and associated phenomena. The answers they provided were firstly that the FRN and related phenomena ***are*** influenced by dopaminergic transmission, although the mechanisms are not yet completely clear. Secondly, there is uncertainty in how DA cells might code negative RPEs and, beyond being inhibited by negative RPEs, there may be serious limits on how sensitively inhibition of DA cell firing can code the size of the negative RPE. However, the leading alternative candidate for the coding of negative RPEs (via serotonergic mechanisms) lacks direct support (see Cools et al., 2011). Thirdly, on grounds of the required rapid temporal dynamics, it is not possible for a change in DA cell firing rate to modulate the medial prefrontal generators of the FRN effect, *at least not via a dopaminergic mechanism*. However, the qualifier of this third conclusion is very important. Ullsperger et al note the studies by various authors (e.g., Chuhma et al., 2004; Lavin et al., 2005; Fields et al., 2007; Hnasko and Edwards, 2012) that show dopaminergic neurons (in the ventral tegmental area) are able to co-release glutamate along with dopamine. As glutamate's action is much more rapid than that of dopamine, the occurrence of co-release offers a non-dopaminergic mechanism whereby firing rate changes of midbrain DA neurons might be able to modulate the FRN, and do so with the rapid temporal dynamics that cannot be achieved by dopaminergic release. Co-release of another fast-acting neurotransmitter (GABA) might be another possible mechanism with the required rapid temporal dynamics (Borisovska et al., 2013; Ullsperger et al., 2014).

Above we have gone into some detail about the use of endophenotypes and our specific choice (the FRN). In sum, and on the balance of the evidence, the FRN is suitable for our DCN framework investigation of the psychobiology of extraversion because this endophenotype is related to learning from reward cues and is likely to be affected by firing rate changes of midbrain dopaminergic neurons, albeit in a complex way. Moreover, we can simulate these DA cell firing changes via a biologically-anchored computational model which is described below. We now move on to step 2 of the DCN framework and review the evidence that the FRN is linked to extraversion.

### Step 2: gather evidence to test whether the endophenotype and E are associated/correlated

On the basis of the evidence reviewed above we decided to investigate the relationship between extraversion and the FRN. At the time we carried out our first study (c. 2009) there was a small number of studies testing the associations between various personality trait measures and ERP phenomena similar to the FRN. An important study was by Boksem et al. (2006) who found that the error-related negativity (ERN; a similar, but earlier, component to the FRN) was significantly related to Carver and White's (1994) BIS scale, and the error positivity (another component) was significantly related to Carver and White's BAS scale. They also reported trends (*p* < 0.1) for relationships with Extraversion for both these components. These authors extended these findings and were able to replicate these associations only under specific reinforcing conditions (Boksem et al., 2008). Similar ERN measures had previously been found to be associated with Neuroticism (see Boksem et al., 2006, for references) and in some of these studies the relationship with Extraversion was non-significant.

Data relating extraversion and the FRN were of considerable interest and so we thus carried out two studies in our lab to address this issue, using an associative reward learning task developed by Potts et al. (2006). This task offers a very simple method for eliciting an FRN that varies across task conditions, as it required no overt responses and thus allowed computational modeling to proceed in a very straightforward way.

In this task a sequence of two stimuli (S1 and S2) was followed by a small cash reward or not. The task (designed to resemble a slot machine game, but requiring no responses) was designed to measure the FRN during S2. Participants simply watched the stimulus sequences and accrued rewards accordingly. An example of a sequence of stimuli on a particular trial is shown in Figure 2, upper panel. S1 can either be a gold bar or a set of lemons. S1 is followed 80% of the time by the same stimulus at S2 (and on the other 20% of the time by the other stimulus). The stimulus shown at S2 determines the trial outcome 100% reliably (if S2=gold bar this predicts a small cash reward; if S2=lemons this predicts no reward). This arrangement of stimuli means that S1 stochastically predicts S2, and also stochastically predicts the delivery of reward on a particular trial. Thus, psychophysiological reactions (in the form of the FRN) to the confirmation or violation of these expectations can be measured at S2, independent of the trial outcome itself. There are 4 classes of trials: predicted reward (PR; S1=S2=gold bar); predicted non-reward (PNR; S1=S2=lemons); unpredicted reward (UR; S1=lemon; S2=gold bar); and unpredicted non-reward (UNR; S1=gold bar; S2=lemons).

The FRN in this task is recorded in the interval 200–300 ms after S2 onset, as indicated in Figure 2 (lower panel). The waveform is usually referenced to a baseline period: the 100 or 200 ms prior to S2 onset in this task. The waveforms in the figure (redrawn from Potts et al., 2006) show that the most negative FRN occurs for the unexpected non-reward trials with the least negative being for the unexpected reward trials. In fact, this waveform (for the unexpected reward trials) can sometimes be a positive deflection (Potts and colleagues have sometimes referred to it as the P2a in honor of its positive sign). This ordering of the waveforms has been replicated in a number of different studies (e.g., Martin et al., 2009). In a combined fMRI and ERP study with this task (Martin et al., 2009) evidence showed that either the ACC or caudate (another target of dopaminergic projections) were potential sources of the FRN; moreover, the haemodynamic fMRI responses in the ACC during the associative reward learning task showed the same ordering of responses as that revealed by the ERPs shown in Figure 2. By using this task, and studying its relationship with extraversion, we have been able to carry out step two of the DCN framework.

In our lab (Smillie et al., 2011), we used the Potts associative reward learning task and tested a group of extraverts and a group of introverts; these extreme groups were selected from a sample who had completed the revised Eysenck Personality Questionnaire and scored more than 1 *SD* above or below the sample mean on extraversion. The groups differed by an average of 15 points (the groups' mean extraversion scores were 21 and 6) on the 25 point extraversion scale. We also replicated the typical ordering of FRNs in the task, although the waveform in response to unexpected reward trials was not positive (in contrast to Figure 2). As in previous studies with this task, the largest difference was between the responses to unexpected trials (unexpected reward minus unexpected non-reward) and we used this “difference FRN waveform” as our key endophenotype. We construe this as an electrophysiological index of the difference between RPE effects (a positive RPE effect minus a negative RPE effect). The difference FRN waveform was significantly larger for our 15 extraverts relative to our 15 introverts.

Our group has recently completed a partial replication of the Smillie et al. (2011) study (Cooper et al., 2014). We used the Temporal Experience of Pleasure Scale (TEPS; Gard et al., 2006) as the main personality measure in this second study. This scale is designed to capture, in separate measures, the anticipatory and consummatory facets of pleasure. The TEPS showed parallel findings to those reported above for extraversion: the difference waveform (FRN for the unexpected reward minus the FRN for the unexpected non-reward) was significantly and positively correlated with the TEPS-Anticipatory subscale (*r* = 0.39, *p* = 0.017), but not with the TEPS-Consummatory subscale (*r* = 0.11, *p* = 0.519). This was not an extreme groups design and we had measures on other scales too, including a measure of EPQ-R Extraversion. Using this measure we broadly replicated our earlier findings: the correlation between E and the (UR-UNR) FRN difference waveform was statistically significant (*r* = 0.36). In addition, other scales (such as EPQ-R Neuroticism and the Carver and White BIS scale) showed no significant relationship with the RPE effect, as measured by the FRN difference waveform.

Based on the data reviewed above, we concluded that the FRN measures from the Potts et al task satisfied step 2 of the DCN framework. If we were to stop at this point, we might briefly consider what these studies with the FRN and extraversion could tell us about the processes that may underlie extraversion. We could conclude that extraverts have higher electrophysiological responses to discrepancies between reward expectations and reward outcomes (RPEs), and that this might arise because extraverts have differences in neurotransmission within the brain circuitry, including midbrain DA neurons, which influences the size of these electrophysiological signals in response to RPEs. In terms of theories about the basis of extraversion, these FRN data are very broadly consistent with long-standing theories that link extraversion to “reward processing” (in some way), and with theories that suppose that extraversion has a partly “dopaminergic basis” (of some kind). These are very general conclusions that, in our view, don't really extend the general theories other than by adding further data that are broadly consistent with them. By applying the next steps of our DCN framework, we attempt to show below how this limited understanding of the possible psychobiological basis of (aspects of) extraversion might be refined and tested, and thus generate sharper predictions for future studies.

### Step 3: use cognitive neuroscience/computational models, and particularly those which have potentially biologically identifiable parameters, to simulate the target endophenotype

Having chosen our endophenotype, and shown that it is related to extraversion, we next must choose a biologically inspired computational model to simulate the endophenotype. Before doing this we note that few studies have attempted computational neuroscience modeling of individual differences in personality. Pickering and Gray (2001) were the first to use a neural network to model individual differences deriving from personality trait variation. The model was a reward learning model, and they argued that the processes being simulated involved the brain's dopaminergic functions. However, the model used in this work was not strictly an RPE-based model, nor was it successful (see below). For the next decade there was very little similar work although at least one major paper was, in part, stimulated by Pickering and Gray's approach (Read et al., 2010).

Very recently, however, a series of papers have appeared which have investigated personality trait effects using computational models: Brazil et al. (2013) investigated psychopathy-related personality traits using reinforcement learning (RL) models which were fitted to participants performance on a probabilistic decision making task; White et al. (2013) used an RPE learning model jointly with fMRI to investigate the behavior of youths with disruptive behavior disorders while they were performing a probabilistic financially-reinforced object choice task; and Skatova et al. (2013) reported the effects of extraversion on a two-stage probabilistic rewarded choice task designed to separate behavior driven by “model-free” reinforcement learning (of the kind we have discussed to date), from behavior relying on more complex, and flexible, “model-based” reinforcement learning.

The goal of the current paper is to develop further this trend for a computational modeling approach to the study of individual differences, specifically with reference to potential dopaminergic substrates of personality traits. To do this we need to select a biologically-anchored computational model which can capture the effects reported above for the Potts associative reward learning task used to generate FRNs. As noted this model will not simulate the EEG signals of the FRN directly, but the neural modulation of those signals. We do this by simulating phasic changes in the firing of midbrain DA neurons. Based on the evidence reviewed above we must keep in mind that our simulated DA cell firing changes cannot modulate the FRN by DA release at terminals in the ACC where the FRN is generated. Instead, we are following the suggestion made by a number of authors (see above for details) that co-release of another “fast” neurotransmitter (either glutamate or GABA) is a much more likely candidate.

There is a large family of reinforcement learning (RL) models which could in theory simulate the RPEs that would be generated in the varying trial types of the Potts et al task. Many such models equate the RPEs, which they simulate, to phasic changes in midbrain dopamine cell firing, as noted earlier^3^. However, many of these RL models do not say much more than this about the neural processes involved. In order to maximize potential insights from our modeling, and future hypotheses to be tested, we prefer to employ an RL model which has a degree of brain mapping. By doing so the parameters, which we will manipulate below, can be (loosely) equated to more localized and more specific psychophysiological processes. On this basis, we chose an established RL model, published by Brown et al. (1999; henceforth BBG), over other possible candidates. This model gave a localized and quite specific biological account of all the critical model components, unlike many rivals. This model was able to simulate all the classic dopamine and RPE phenomena published at the time, and the authors provide a reasonable and quite detailed discussion of why it did so better than many contemporary alternatives (see the discussion by Brown et al., 1999). There have been few, if any, direct tests of this model, or any of its contemporary rivals since its publication. The basic loops it proposes have broadly stood the test of time, as witnessed by the similarities between the core elements of the BBG model and those of Brown's more recent PRO model (Alexander and Brown, 2011), although some further complexities in the circuitry have been added (see, for example, Bromberg-Martin et al., 2010).

### The brown bullock and grossberg (BBG) model

**Figure 3:**
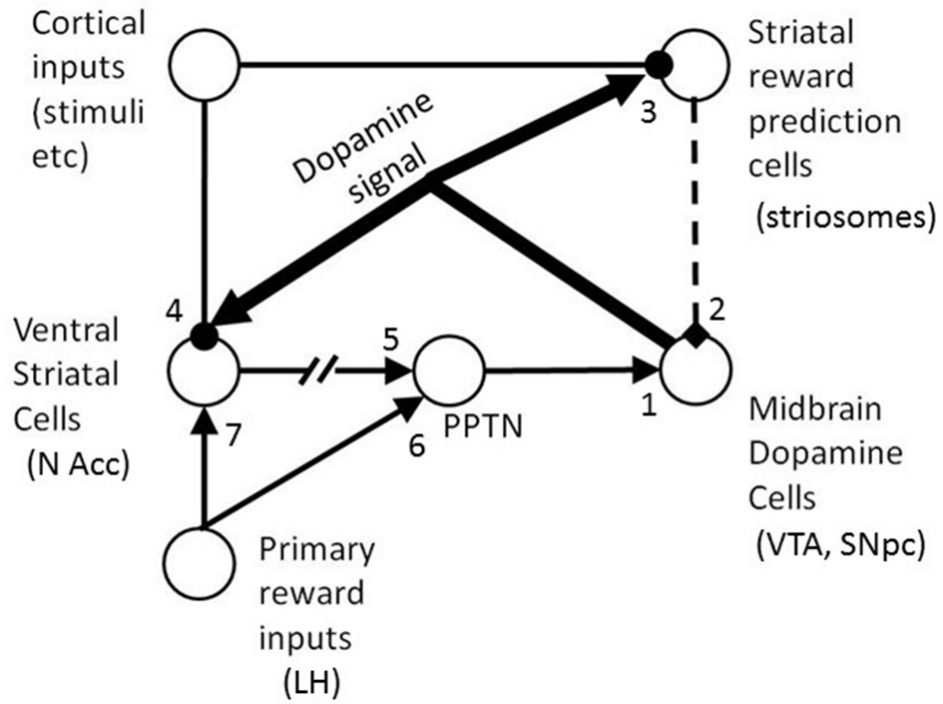
**The basic architecture of the BBG model**. Solid standard thickness lines are excitatory projections; and those with rounded terminals are modifiable by learning. Dotted lines indicate an inhibitory pathway. Very thick lines are ascending neuromodulatory (dopamine) projections. The projection from N Acc to PPTN is broken to show that it is indirect (via ventral pallidum). It is a double inhibitory pathway, but is represented as (nett) excitatory for simplicity. The numbers indicate some of the locations at which individual differences were introduced to the model (see text for details). In the model there is a conditionable pathway (via site 4 then ultimately via site 1) by which cortical stimulus representations can activate the midbrain DA cells. There is also a conditionable inhibitory pathway (via site 3 then site 2) by which the cortical stimulus representations can activate DA cells. BBG, Brown, Bullock, and Grossberg; DA, dopamine; LH, lateral hypothalamus; N Acc, nucleus accumbens; PPTN, pendunculopontine tegmental nucleus; VTA, ventral tegmental area; SNpc, substantia nigra pars compacta.

The basic architecture of the model is shown schematically in Figure 3. The key to the model is the existence of an excitatory reward conditioning pathway and an inhibitory reward prediction pathway which both converge on dopaminergic midbrain cells. The firing of these DA cells changes phasically in response to the balance of their inputs from these two pathways: if the excitatory pathway has the greater influence, then this generates a positive RPE and dopamine cell firing increases; if the inhibitory pathway dominates, then this reflects a negative RPE and dopamine cell firing phasically decreases.

The model also proposed a special spectral timing mechanism; this mechanism enables the striatal striosomal cells, which are learning to predict the occurrence of reward from the stimuli present, to time the occurrence of the reward prediction quite precisely (see below). The phasic changes in firing of the dopamine cells act as a reinforcement signal modulating the weight changes of the plastic synapses in both the excitatory and inhibitory pathways. This learning control by a dopaminergic RPE signal is computed using variants of the three-factor learning rule, popular in many DA based learning models (Reynolds and Wickens, 2002). The three factors refer to the conditions necessary for synaptic change to occur. The first two factors are pre-synaptic and post-synaptic activity. These are the familiar elements of the more basic mechanism of Hebbian learning potentiation (Hebb, 1949). The third factor is a reinforcement signal without which synaptic change will not occur and, in the BBG model as well as many other RL models, timed phasic firing changes in midbrain DA cells provide the RPE signals which serve as the reinforcement signals that complete the 3-factor learning rule.

When a positive RPE occurs the active synapses, in both the excitatory and inhibitory pathways, are strengthened; when a negative RPE occurs the active synapses on both pathways are weakened. Thus, if the prediction of reward is accurate (in terms of occurrence, timing, and amount of reward) there is no RPE and no further learning of reward prediction takes place on the inhibitory pathway. Similarly, when the RPE is zero, there is no further appetitive conditioning (on the excitatory pathway) of any conditioned stimuli present prior to the occurrence of the reward.

In terms of brain structures, the BBG model proposes that the unconditioned reward acts on the lateral hypothalamus (LH) which projects to the pedunculopontine tegmental nucleus (PPTN), and from there via excitatory synapses on to the tegmental midbrain dopamine cells (in the ventral tegmental area, VTA, and substantia nigra pars compacta, SNpc). The reward signal from the LH also projects to the ventral striatum (nucleus accumbens). Medium spiny cells in the accumbens also receive glutamatergic inputs from limbic and cortical cells which encode working memory (WM) representations of the stimuli present. Under the influence of the RPE signal, transmitted via ascending DAergic projections and causing DA release close to the cortico-accumbens synapses, the accumbens cells are thus conditioned to respond to appetitive CSs which regularly precede a reward. The output from the accumbens cells is an inhibitory GABAergic projection to the tonically active cells in the ventral pallidum which in turn sends an inhibitory input to PPTN. Thus, increased accumbens output (in response to unconditioned or conditioned reward), has a nett excitatory effect on PPTN, by inhibiting its pallidal inhibition.

The same cortical cells, which encode WM stimulus representations, also project to reward predicting striosomal cells, in the striatum. Via the spectral timing mechanism noted earlier, each striosomal cell is transiently active for a brief period (10 s of ms) at a distinct and specific time after WM activation. The striosomal cell can learn to predict a rewarding event only when it is active and therefore a specific striosomal cell is tuned to rewarding events at a specific time interval following a stimulus event. Thus, an array of striosomal cells, each with different intrinsic dynamics and therefore different timing properties, can span an extended post-stimulus interval (potentially several seconds in duration). The striosomal array can thus encode a temporally precise prediction of any reward which occurs within the post-stimulus interval. After learning the timed reward prediction, for a particular stimulus, that stimulus then triggers GABAergic output from the striosomal cell array, which inhibits the DA cells. The result is that a stimulus-dependent reward prediction creates a phasic reduction in DA cell firing at the specific post-stimulus time lag at which the reward had previously occurred, in the presence of the stimulus concerned. When the predicted reward actually occurs at this time point, the excitatory pathway leads to a phasic increase in DA cell firing; this increase cancels the decrease created by the reward prediction pathway (and the two pathways can be described as opponent processes). If the predicted reward does not occur, or is smaller than experienced previously, or occurs at a different time post-stimulus, then the inhibitory effect of reward prediction can be observed on the firing of the dopamine cell. If an unpredicted (or under-predicted) reward occurs after a particular stimulus, then the excitatory input to the DA cells is not (fully) canceled out by the inhibitory reward prediction, and so a phasic increase in DA cell firing occurs. These effects are observed in single cell recordings of DA cells in the monkey (see Schultz, 1998, for a review) and are accurately simulated by the BBG model (Brown et al., 1999).

### Step 4: carry out the “individual differences” simulations of the endophenotype (or processes contributing to endophenotypic variance)

The major insights from the current work will flow from considering the issues relating to this step of the DCN. We show below that the relationship between the model parameter and the simulated endophenotype must be *very strong* if the model parameter is to be a plausible source of variation in the phenotype. In this paper, we will refer to this as the *very strong relationship* (VSR) constraint on our candidate biological parameters. In addition, we will argue that the amount of variance in the simulated output (our endophenotypic process) must be also be as large as possible; again the reasoning for this will be made explicit below. We refer to this as the *maximal variance* (MV) constraint.

In the case of a simple computational model, with few parameters, one could estimate the specific parameter values which best capture the measured behavior of an individual participant on a specific task (the endophenotype in question). Then, one could see if the values of one or more of these estimated parameters were correlated, across individuals, with the scores for the phenotype of interest (in this case a specific personality trait). Significant correlations of this kind would be consistent with the hypothesis that the process, reflected by the model parameter, is part of the causal basis for the personality trait concerned. Brazil et al. (2013) refer to this approach as seeking the “computational phenotypes” of a personality trait or psychopathological condition (i.e., attempting to quantify the latent process[es] which characterize the traits, or conditions, of interest).

With more complex models, with larger number of parameters, this “direct” approach cannot easily be pursued, as estimating the best fitting model parameters required to capture an individual's endophenotypic behavior will usually not be possible. In this article we consider what can be achieved with the DCN approach applied to a much more complex computational model; the complexity of which allows it to be brain-mapped to potential neurophysiological processes of interest.

The DCN framework is a general approach that can be employed with any computational model as a means of simulating individual differences in the model outputs. In the present paper we will apply the DCN framework to the BBG computational neuroscience model (Brown et al., 1999) which we used to simulate the modulatory signals affecting the FRN. However, we could adopt the DCN framework with whatever specific model we were using and whatever endophenotype we were simulating. To simulate individual differences, we systematically replace the constant values typically used for model parameters, with random variables. Our goal is to see what effect each model parameter has on model outputs.

In one sense, all model parameters are relevant to the outputs simulated by the model, or else they could be eliminated from the model. However, in specific modeling scenarios, some model parameters can have a much greater effect on the simulated outputs than others. For example, we observed relatively weak relationships between some model parameters and simulated outputs in our first published work of this kind (Pickering and Gray, 2001). In that study we argued that a key role of dopaminergic neurotransmission was in providing a reinforcement learning signal. We constructed a very simple reinforcement learning model (but not strictly an RPE-based model). This model used the reinforcement signal in simulations of a simple category learning task. In published studies, learning performance on the category task was significantly associated with sensation seeking trait measures (Ball and Zuckerman, 1990; high sensation seekers learned the task faster than low sensation seekers). In our modeling, we allowed the value of the reinforcement signal (which was used to drive learning following a correct simulated response) to vary across simulated individuals. We expected a negative correlation, between the size of the reinforcement signal used and the number of simulated trials that were required to learn the task to a particular criterion level of success. However, to our surprise, the correlations we obtained were very modest. This finding held across a very wide range of modeling variations that we tried (−0.35 < *r* < 0). We therefore concluded that variation in the reinforcement parameter across individuals was not a plausible basis for the reported association between sensation seeking personality and learning rates in this task. We reached this conclusion because the relationship between model parameter and simulated endophenotype was, by our reasoning, almost certainly not strong enough to underpin the observed trait-endophenotype correlations in the real data. This parameter (the dopaminergically-mediated reinforcement learning rate) therefore failed the “VSR constraint,” noted earlier. Below, this VSR constraint will emerge as a key test in the current simulations which we use to discriminate between plausible and implausible sources of covariance between the endophenotype and the phenotype.

In the DCN framework, as just stated, we deliberately create systematic variance across simulated individuals by allowing model parameters to vary as a random variable. A convenient default choice is to allow model parameters to be replaced by random normal variables; this is equivalent to assuming that each model parameter is normally distributed across (simulated) individuals. Without more specific information to inform this choice, assuming a normal distribution seems to be a reasonable starting point. As we are going to try to equate model parameters to specific psychobiological processes, this modeling assumption just implies a (default) belief that these psychobiological processes are normally distributed across individuals.

Some of the causal processes that we are interested in are possibly single genetic variants. These would not be expected to be normally distributed across individuals: such genetic variations are more likely to cause a step change in a specific neurotransmission-related process. It might therefore seem as if these would be better modeled by setting a parameter to, for example, a high vs. a low value. However, if there were non-linearities in the relationship between parameter and simulated process affecting the endophenotype, then any specific pair of high vs. low parameter values could either produce, or not produce, an effect in the simulations. This would depend on the specific values chosen and how they are positioned relative to the non-linearities in the parameter-process relationship. So, even in this case, by using a range of parameter values (e.g., based on a normal distribution) one can get a clearer picture of the effect of a parameter on the process being modeled.

It follows from the foregoing that, for a particular model parameter, a simulated individual will be assigned a particular parameter value that is specific to them (and constant throughout their individual simulation); this individual parameter value is drawn from a random (normal) distribution of parameter values with specified distributional properties (e.g., a mean and variance). The key results, of the kind reported below, are assessments of whether there is a relationship, across a sample of simulated individuals, between model parameter variation and simulated endophenotypic process (as captured by a specific model output). Another key result is the amount of variance in the simulated output that is created by adding variance to a particular model parameter.

**Figure 4:**
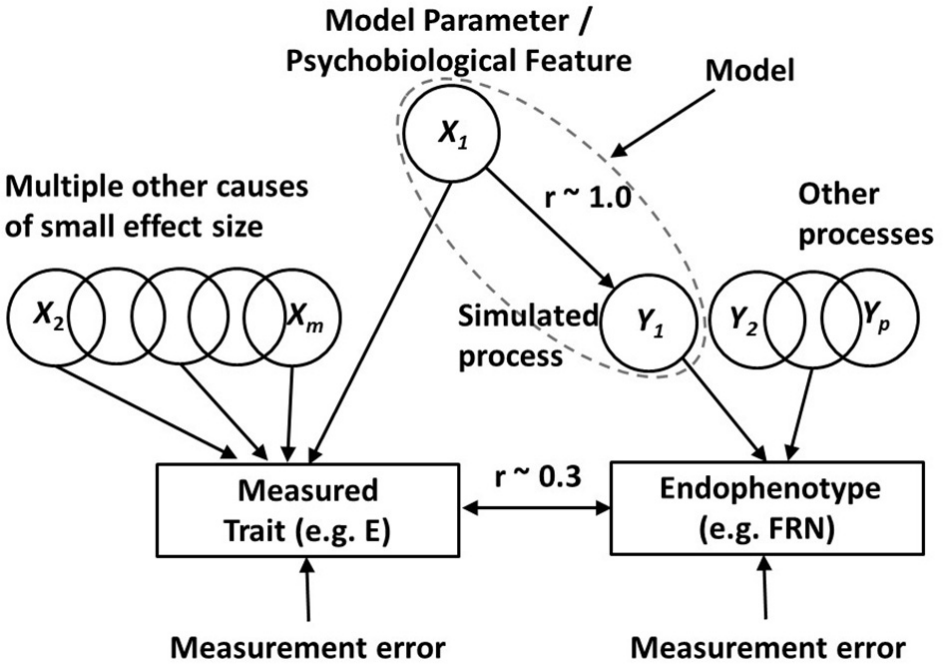
**The differential cognitive neuroscience (DCN) modeling framework**. In the model being deployed (dotted ellipse) there are multiple parameters, such as *X*_1_, which can be manipulated as potential sources of individual differences. This highlighted parameter in the model putatively captures a psychobiological feature (such as dopamine receptor density in a particular brain region) which is a candidate source of variance in a phenotypic trait of interest (such as Extraversion, E). The framework is used to investigate whether the psychobiological feature is a plausible source of variance in the trait. It is assumed that there are also multiple other sources of trait variance (*X*_2_ to *X*_*m*_), outside the model. The figure shows an endophenotype (such as the feedback related negativity, FRN) that is known to be related to the trait of interest. There are assumed to be a number of processes, *Y*_1_ to *Yp* (*p* < *m*), which contribute variance to the endophenotype. One of these processes (*Y*_1_) is being simulated by the computational model that includes parameter *X*_1_. When (as depicted here) the psychobiological feature, reflected by the simulation parameter *X*_1_, is a source of covariance between phenotype and endophenotype, it is demonstrated (see text for details) that the relationship between *X*_1_ and simulated process *Y*_1_ needs to be very strong (*r* ~ 1) in order to be able to see a typical correlation of *r* = 0.3 between the phenotype and endophenotype in real data. If a model parameter fails to exert a strong influence on the simulated process, then the psychobiological feature, represented by the parameter, cannot be a plausible candidate for the observed phenotype-endophenotype association.

In the DCN framework the simulations start by adopting what will be referred to as the *noise-free* version of the computational model. This version, as the name implies, does not have any significant exogenous sources of noise in the process being simulated. We do this so that we can see the effects of our systematic parameter variation against a backdrop of little or no random noise in the simulations. By using a noise-free model (almost) all the variance in the model output, across simulated individuals, must therefore be derived from the variation in a model parameter, which was imposed, across simulated individuals. Figure 4 illustrates how the noise-free model sits within the broader DCN framework.

In using the BBG model for the simulations we will manipulate a series of psychobiological parameters from the model, one of which is labeled as *X*_1_ in Figure 4. In the noise-free BBG model simulations, variation in parameter *X*_1_ will be the primary source of variation in the process being simulated by the model, which in the present case is phasic changes in the firing of the DA cells (this process is denoted *Y*_1_ in Figure 4). *Y*_1_ is thus the key model output and is assumed to be a substantive component of the endophenotype that we are trying to model (the FRN in the present case). In the present paper process *Y*_1_ (phasic firing changes in midbrain DA neurons) exert a modulatory influence on the FRN, which is generated by neurons in medial prefrontal brain areas such as the ACC. The phasic firing changes occur in response to the RPEs generated by the various conditions of the specific FRN task that we are simulating (the Potts et al task associative reward task, described in detail above).

Figure 4 shows a number of parameters like *X*_1_ which all contribute to variance in extraversion (they are labeled *X*_1_ to *X*_*m*_), and a number of processes like *Y*_1_ contributing to the endophenotype (they are labeled *Y*_1_ to *Y*_*p*_). In keeping with the view that each causal process has a greater influence on the endophenotype than on the phenotype, we assume that *m* is a larger number than *p*. This assumption means that the psychobiological parameters in our computational model have a stronger relationship with the processes underlying the simulated endophenotype than they do with personality traits like extraversion.

Figure 4 also shows the VSR constraint: we are looking for simulation parameters (*X*_1_) which have a very strong relationship with the simulated process (*Y*_1_) affecting the endophenotype. Figure 4 shows that the correlation between *X*_1_ and *Y*_1_, required under this constraint, is approaching 1.0. We argue that this VSR is required in order for the observed relationship between the trait (phenotype) and endophenotype to be of the magnitude that is typically observed in real data (shown as *r* ~ 0.3 in Figure 4). The correlation between the phenotype and the endophenotype results from the shared cause(s) influencing each of them. In Figure 4 there is only one shared cause: the modeled biological parameter *X*_1_. This is shown as affecting the endophenotype via the process *Y*_1_. Therefore, the correlation between the phenotype (trait E) and the endophenotype (the FRN difference wave) will be determined by the geometric mean of two variance ratios, A and B (i.e., $\sqrt{A^{*}B}$); A is the variance of *X*_1_ divided by the total variance of the trait, and B is the variance of *Y*_1_ due to *X*_1_, divided by the total variance of the endophenotype. As we have already noted this correlation will often tend to be quite modest. This is because the variance ratio A gets progressively smaller as more independent causal factors contribute to the trait variance, i.e., with increasing *m* in Figure 4. We have already suggested that we believe *m* is large for personality traits. Similarly, the variance ratio B gets progressively smaller as more independent causal factors contribute to the endophenotype, i.e., with increasing *p* in Figure 4. Even if we (unrealistically) assumed that only 1 process affects the phenotype (*p* = 1) plus measurement error, then with multiple independent processes contributing equally to trait variance, the expected trait-endophenotype correlation will be small.

To show the above prediction concretely, we can make an arbitrary (but probably conservative) assumption that *m* = 8. If the reliabilities of the trait and the endophenotype were 0.8 and 0.9 respectively, and the correlation between *X*_1_ and *Y*_1_ was perfect, then *A* = 0.1, *B* = 0.9, and the trait-endophenotype correlation would be 0.3 (i.e., in the typical ballpark for real data). Clearly, with a single shared causal process, this trait-endophenotype correlation would be even more modest if *m* were greater than 8 and/or *p* were greater than 1. If the correlation between *X*_1_ and *Y*_1_ were less than perfect the trait-endophenotype correlation would also drop dramatically. For example, if the correlation between *X*_1_ and *Y*_1_ were 0.5 this means that the proportion of *Y*_1_ variance due to *X*_1_ is 0.25. In the above calculations, the variance ratio B would reduce from 0.9 to 0.9^*^0.25 = 0.225, and the trait-endophenotype correlation would therefore drop correspondingly to 0.15.

However, satisfying the VSR constraint is only necessary, but not sufficient, for identifying plausible parameters underlying the phenotype-endophenotype correlation. Earlier we suggested that another important constraint applies: we referred to it as the maximal variance (MV) constraint. This just means that the variance added to a single parameter in our noise-free model must create as much variance as possible in the key output being simulated by the model. Again the simple psychometric arguments made above show why this is important. In Figure 4 a psychobiological feature, reflected by a varying parameter (*X*_1_) in our model, is a source of variance in our phenotype (extraversion) and also drives the resulting process (*Y*_1_; DA firing rate differences across different trial types in the task) that contributes variance to our endophenotype (the FRN difference wave from EEG data). Above we argued that the correlation between the endophenotype and the phenotype is given by $\sqrt{A^{*}B}$), where *B* is the variance of *Y*_1_ due to *X*_1_, divided by the total variance of the endophenotype. The VSR constraint is a requirement that almost all the variance of *Y*_1_ is due to *X*_1_; however, in order for the ratio *B* to be as large as possible (and therefore maximize the phenotype-endophenotype correlation of interest), it is clear that process *Y*_1_ needs to have as large a variance as possible. If the variance in *Y*_1_ is small, even though it mostly derives from X_1_, then it will be swamped by the other sources of variance affecting the endophenotype (*Y*_2_ to *Y*_*p*_ in Figure 4). If *Y*_1_ variance were small relative to these other sources, then the ratio *B* would be small, and the correlation between endophenotype and phenotype would be likely to be undetectable.

The approach to be used in this paper is to investigate the influence of variation in many of the parameters in the model, with each parameter's influence being tested on its own. This approach is close to a process, used in various fields, known as sensitivity analysis (Parnell, 1997). By the logic above, the parameters to which the size of the simulated endophenotypic process (the simulated DA cell phasic firing changes) is most sensitive are those which are the more plausible candidates for underlying the observed relationship between extraversion and the FRN. By using a biologically-mapped model, we are then able to try to give a psychobiological interpretation of the plausible (and implausible) parameters, and in particular how they might relate to neural processes linked to dopamine cell firing.

The important background issues, which we have considered in the preceding paragraphs, have set the scene for us to carry out Step 4 in our DCN modeling; the details of this how this step was carried out, and what outcomes were obtained, are reported in the Methods and Results Sections below. Steps 5 and 6 will be briefly explored in the Discussion.

## Methods: basic simulation details

In this first methods section we summarize (in conjunction with Supplementary Material) the basic computational methods used in the simulations.

### Using the BBG model to simulate the Potts associative reward prediction task

Having selected the BBG as our specific RPE model, the goal was to change it as little as possible from the published version, in order to enable simulations of the Potts et al associative reward learning task. At the same time we wanted to preserve the ability of the model to simulate the tasks captured in the original Brown et al. (1999) paper. However, a small number of minor changes were required. The working memory (WM) representations of the conditioned stimuli used in the original paper were not realistic (these were coded as square wave signals starting at stimulus onset and ending immediately at an arbitrary time thereafter). A particularly unrealistic result of this WM coding occurs whenever the same stimulus was presented twice in succession with a blank (or fixation) screen between the two presentations (as occurs in the Potts task on the majority of trials). When this happens, and if the WM representation triggered by the first occurrence of the stimulus was still active when the second stimulus presentation occurred, there would be no change in WM representation and therefore nothing that signifies that two separate presentations have occurred.

A change was therefore needed in the current implementation. One possibility was to turn off the WM representation of the first presentation of the stimulus during the blank screen before the second presentation occurred. However, although this would allow the two presentations to be distinguished in WM, it would have the unwanted consequence that the prediction of the later stimulus by the earlier stimulus could not be learned (as this requires an active WM representation of the first stimulus at the time when the second stimulus occurred). In the current version of the BBG model, we adopted a different solution in which each stimulus triggered the activation of a WM representation, with an output *x_i_*. The change in this output over time was specified according to the following equation:

(1) $$\frac{1}{\tau_{x}}\frac{dx_{i}}{dt}=\left(M_{x}-x_{i}\right)*\;I_{i}-A_{x}\;*\;x_{i}$$

where each stimulus (coded with a subscript *i*) is represented with a different node in the model, and *I*_*i*_ is a square wave (taking a value of 1 when the physical stimulus *i* was present, and a value of 0 when the stimulus was absent). The values of the parameters in the above equation (*M*_*x*_ = 1; τ_*x*_ = 30; *A*_*x*_ = 0.02) were chosen so that the outputs from the WM representations decayed partially during the interstimulus intervals between the stimuli in simulations of the Potts task. The variable *x*_*i*_ above in our equation (1) above replaced the variable *I*_*i*_ in the original BBG model (in Brown et al., 1999, Equations 1 and 2). Note that, when the stimulus was present, *I*_*i*_ took a value of 0.6 in the original model and, as noted, *x*_*i*_ was set to 1.0. This value determines the value of another parameter in the model, Γ_G_ (see the parameter value table in Supplementary Material). Γ_G_ took a value of 1/(1 + 0.6) – 0.05 in the original BBG model (= 0.37), and a value of 1/(1 + 1.0) – 0.05 in our simulations (= 0.495).

In the BBG model, output from the WM representations can activate the spectral timing mechanisms in striosomal cells that predict future rewards. In the current implementation, activation of spectral timing in the striosomes occurred whenever the output from the WM representation, *x*_*i*_, exceeded a threshold θ_*x*_ = 0.3. Thus, our model computed a variable *xx*_*i*_ which was given by

(2) $$xx_{i}=\max\left(x_{i}-\theta_{x},0\right)\text{ \& }1$$

Thus, *xx*_*i*_ takes a value of 1 when the threshold was exceeded, 0 otherwise. The variable *xx*_*i*_ replaced the variable *I*_*i*_ in the original BBG model (in Brown et al., 1999, Equation 10).

There were other minor changes to the equations used in the original BBG model; these changes are covered in Supplementary Material, where the full set of the parameter values used in the current simulations is also given.

## Results

### Basic modeling results

**Figure 5:**
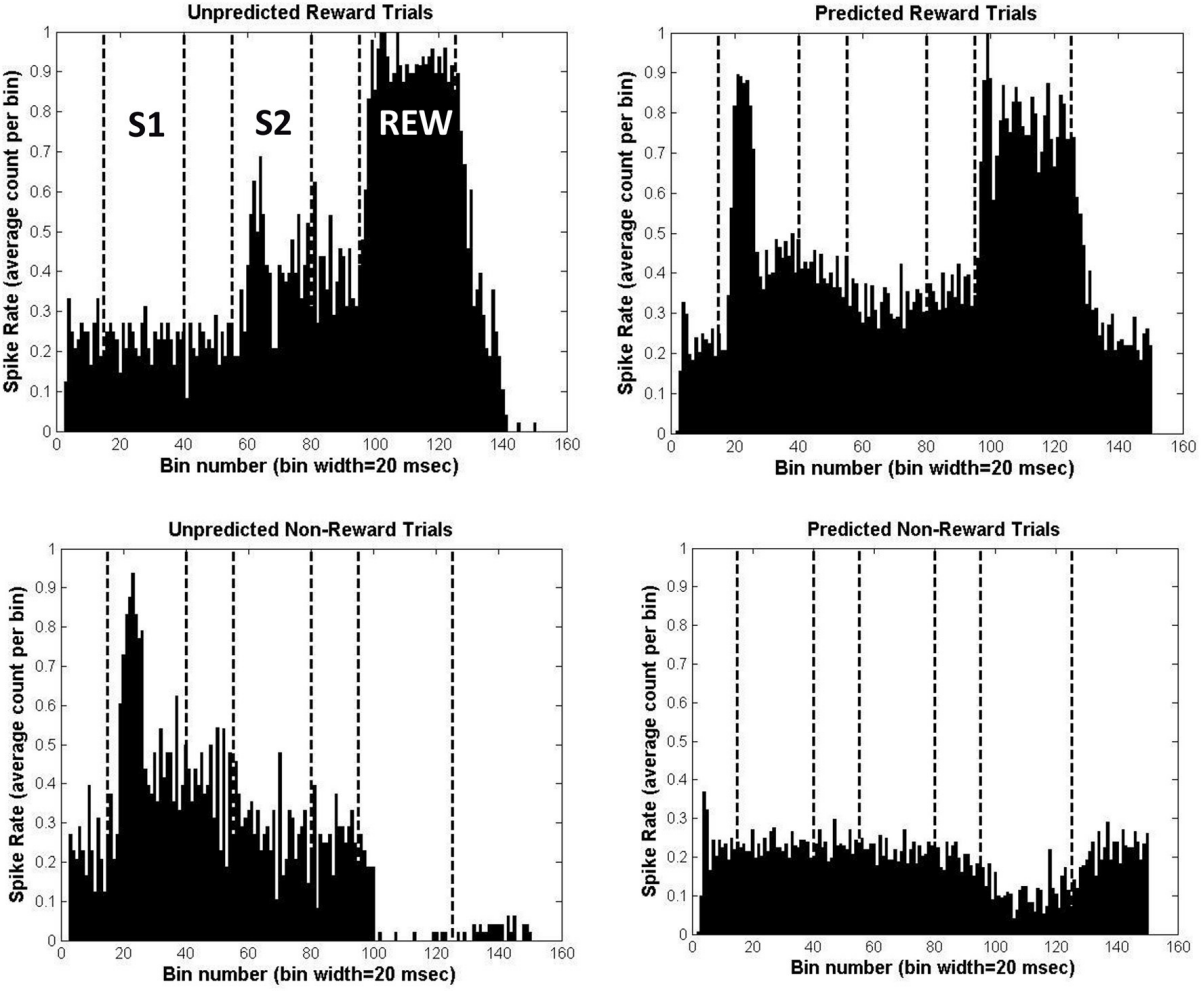
**Simulation results for the Potts associative reward learning task, for a simulated individual with all parameters set to their mean values**. Each panel shows the average spike firing rate for simulated midbrain DA neurons across a trial in the task, divided into 20 ms bins. In the upper left panel are the results averaged across the 48 unpredicted reward (UR) trials; in the lower left panel are the result averaged across the 48 unpredicted non-reward (UNR) trials; in the upper right panel are the results averaged across the 196 predicted reward (PR) trials; and in the lower right panel are the results averaged across the 196 predicted non-reward (PNR) trials.

As a first step, we need to show that the BBG model can capture the patterns of DA cell firing which might underlie the FRN effects in the Potts associative reward learning task. The FRN was recorded during the presentation of the second stimulus (i.e., at the time point denoted S2; see Figure 1 upper panel). Each panel of Figure 5 represents the simulated DA cell firing rates from a single simulated participant, plotted across the duration of a trial. The rates are the averages across all the trials of each of the 4 specific trial types of the Potts associative reward learning task. The times of the key events in a trial (S1, S2, REW) are marked by vertical lines. The 4 types of trials in the task are each shown in a separate panel of Figure 5: unpredicted reward (UR; S1=lemons; S2=gold bar; upper left); unpredicted non-reward (UNR; S1=gold bar; S2=lemons; lower left); predicted reward (PR; S1=S2=gold bar; upper right); and predicted non-reward (PNR; S1=S2=lemons; lower right).

For the UR trials one can see that there is no increase in DA firing during S1, but a strong increase at the time of delivery of the reward (REW). However, the stimulus appearing at S2 on such trials (gold bars) is associated with reward on most trials of the task, and so undergoes some learning of this association (at location 4, in the model; see Figure 3). Thus, the gold bar stimulus occurring at S2 on UR trials elicits a moderate increase in DA cell firing during S2 via the model's excitatory pathway. Given that the FRN was recorded during the S2 stimulus period, this modeled increase in DA firing during S2 might be expected to modulate the size of the FRN during S2^4^.

For the PR trials, one can see that the increase in DA firing at the time of reward delivery (REW) is robust but somewhat weaker than the corresponding DA firing during REW for UR trials. This is because additional reliable inhibitory predictors of reward (gold bars at S1 as well as at S2, compared with just gold bars at S2 for UR trials) have been established. These timed inhibitory associations are learned (at model location 3; see Figure 3) over the course of the task. The earliest reliable predictor (a gold bar at S1) also produces a marked increase in DA firing during S1 via the excitatory pathway of the model. This result is consistent with animal studies in which the earlier of two predictors of a reward triggers DA cell firing increases (see Schultz, 1998, for details).

For the UNR trials we see that the stimulus presented at S1 triggers a strong increase in firing, via the excitatory pathway of the model. This occurs, just as in the PR trials, because the S1 stimulus on such trials (gold bar) has become associated with reward delivery during the task. During REW, there is a strong suppression of DA firing to well below baseline levels. Reward is expected on these trials (based on the fact that 80% of the time when the stimulus at S1 is a gold bar then a reward will be delivered) and there is a corresponding learned inhibition of DA firing occurring at location 3 in the model (see Figure 3). This inhibition is triggered in response to the presentation of a gold bar during S1. The spectral timing properties of the BBG model (see above) mean that the inhibition effect occurs at specific striosomal sites that have the appropriate intrinsic timing properties: i.e., they become active (when stimulated by a stimulus at S1) at a specific time after S1 which corresponds with the timing of the reward. Thus, the inhibition effect is timed to occur when the reward is expected to occur (i.e., at REW). Normally, this inhibition would serve to counteract the boost to DA firing triggered by the expected reward via the excitatory pathway in the model; we can see the inhibitory effect in isolation on these UNR trials because reward is not delivered.

In a similar way, during UNR trials, one can also see inhibition of firing during S2 (relative to the pre-S2 time period), when the unexpected lemon appears. This inhibition is less marked than the inhibition observed on these trials during REW. On 80% of occasions when a gold bar is presented at S1, a gold bar is also presented at S2. As the gold bar stimulus has acquired the ability to trigger DA release via the excitatory pathway of the model (see in UR trials), the usual experience would be for some DA firing increase at S2. However, the nature of the BBG model means that learned inhibition of that DA firing increase at S2 will also occur, in response to the gold bars presented at S1. This also occurs at location 3 in the model (see Figure 3), but occurs on cellular sites with different intrinsic timing properties from the cells which deliver the inhibition that is timed to occur during REW. Normally, this inhibition would oppose the increase in DA firing during S2 occurring when the gold bar occurs at S2 (see the panel for the UR trials). However, on UNR trials we can see the inhibitory effect because the unexpected lemons, occurring at S2, do not trigger DA firing. This suppression of DA firing at S2 on these UNR trials would be expected to modulate the FRN recorded during S2; and the modulatory effect caused by this suppression of DA firing would be in the opposite direction to the modulation resulting from the DA firing increase observed at S2 on UR trials. Thus, the maximal difference in DA firing rates during S2 is observed when comparing the UR and UNR trials, in the same way that the maximal FRN difference is observed for these same two trial types (see Figure 2). Note that although the “UR minus UNR” DA firing difference is greatest during the period when the reward was delivered, the same pattern of differences is also observed during S2, albeit that the differences are less marked. The design of the Potts task deliberately allows the FRN to be observed at a time point (S2) that is removed from the point of reward delivery.

Finally, we might note that the PNR trials show relatively little fluctuation in DA firing, although there is some suppression during the period when the reward is delivered. At first glance, this suppression might be considered a surprising result given that the lack of reward is expected (as lemons had been presented on these trials at both S1 and S2). However, the task involved 48 UR trials in which a lemon at S1 was associated with later reward. These trials thus create a modest amount of learned timed inhibition of DA firing, triggered by the presentation of a lemon at S1. We can see the effect of this inhibition on the PNR trials.

The data in the 4 panels of Figure 5 were each collapsed to give overall normalized DA firing rates during S2 as follows: for each trial type separately, we took the average of the first 300 ms after S2 onset minus the average of simulated 100 ms prior to S2. The results, measured in mean spikes per 20 ms bin and in the order UR, UNR, PR, PNR, were: 0.1506, −0.0664, −0.0594, −0.0113. Note that the DA firing rates increased most for UR trials and decreased most for UNR trials, with the values for PR and PNR trials lying in between. This parallels the order of the FRN waves recorded for these trial types in real data obtained with the Potts associative reward task.

## Methods: adding individual differences to the simulations

In this methods section we summarize the ways in which individual differences in model parameters were employed in the simulations. Our key simulated process (*Y*_1_ in Figure 4) is the difference in DA cell firing for UR minus UNR trials during the S2 phase of the task. We argue that this is a major component of variance in the endophenotype (the FRN difference during S2 between the same two trial types).

In our DCN approach we added random variation to several parameters (*X*_1_ in Figure 4) of the BBG model, one at a time, to create individual differences. Next, we consider some of the possible parameters to which variance was added, and suggest psychobiological interpretations of these parameters. The key outcome across these simulations was what effect this added parameter variance had on the critical simulated process (the difference in simulated DA cell firing between trial types).

The first parameter explored was parameter *w_PD_* (see Supplementary Material). This parameter sets the weight from the PPTN to the midbrain DA cells. This parameter is at location 1 in Figure 3, and is the final common path on the excitatory route to activation of the DA cells. It modulates the size of the total excitatory drive coming in to the DA cells, by controlling the strength of the projection from PPTN to midbrain DA cells. This is a particularly important parameter to explore, because it could be said to offer the most direct test of the so-called reinforcement sensitivity theory (RST) of extraversion. This theory, originally proposed by Jeffrey Gray, suggests that extraversion may derive partly from individual differences in sensitivity/reactivity to rewarding stimuli, both conditioned and unconditioned rewards (see Smillie et al., 2006, for a review). Under this theory, an extravert is someone who has stronger than average reactivity to rewards. Our research with the Potts task showed that extraverts have a larger FRN difference wave (comparing UR trials minus UNR trials) than introverts. Thus, we can use RST to make a specific prediction here: simulated individuals with high settings for *w_PD_* will have a high DA cell reactivity to rewarding stimuli and thus, according to RST, should be “simulated extraverts.” Larger DA cell firing rate differences between UR and UNR trials in the Potts task are expected to equate to larger FRN differences between UR and UNR trials and so should be a characteristic of extraverts. Thus, the DA cell firing rate differences should correlate positively with *w_PD_*.

Next we will vary a series of other parameters in the model and the findings from these simulations can be compared with the findings from the “benchmark” *w*_*PD*_ simulations noted above. We will begin by looking at parameters on the excitatory model pathway of inputs to the DA cells and then move on to parameters on the inhibitory pathway of the model.

For each of these simulations, 50 simulated individuals were created using a random normal variable to replace the fixed value of a specific parameter. The key simulation output is derived from normalized simulated DA firing rates. The rates are computed for the first 300 ms of the simulated period when stimulus S2 was presented to reflect the timing of the FRN; the rates were normalized relative to the 100 ms preceding S2; then the rate is averaged across the 48 UR and 48 UNR trials for each simulated individual, and the key output is the difference in mean normalized firing rate (for UR trials minus UNR trials). This output reflects the simulated process that theoretically should contribute variance to the FRN difference wave (for UR minus UNR trials) recorded in real electrophysiological data. The units of the simulation output are DA spikes per 20 ms recording bin.

## Results and discussion

### Individual differences simulations

Table 2 gives the results of simulating individual differences. The table lists representative results for simulations manipulating various parameters, detailing summary information for the key simulation output in each case.

**Table 2:** **Typical results from individual differences simulations**.

**Parameter varying (and location in Figure 3)**	**Parameter mean (population, [sample])**	**Parameter *SD* (population, [sample])**	**Simulation output, sample mean**	**Simulation output, sample *SD***	**Correlation (parameter, simulation output)**
**AFFECTING MODEL EXCITATORY PATHWAYS**					
*w*_PD_ (1)	5 [5.116]	1 [1.003]	0.225	0.052	0.98
*w*_PD_^*^ (1)	5 [4.838]	1 [0.824]	0.214	0.041	0.95
*w*_SP_ (5)	2 [2.058]	0.5 [0.501]	0.230	0.080	0.98
*w*^‡^_SP_ (5)	2.125 [2.125]	Range 0.25–4.0	0.239	0.156	0.99
*w*_RP_ (6)	0.8 [0.823]	0.2 [0.201]	0.228	0.011	0.91
*w*^‡^_RP_ (6)	2.05 [2.05]	Range 0.1–4.0	0.269	0.041	0.97
*w*_RS_ (7)	1.5 [1.540]	0.35 [0.351]	0.216	0.020	0.59^†^
*w*^‡^_RS_ (7)	1.05 [1.05]	Range 0.1–2.0	0.165	0.069	n/a
*w*^*max*^_*s*_	1.0 [1.029]	0.25 [0.251]	0.225	0.042	0.92
τ_WS_ (4)	1 [1.028]	0.25 [0.25]	0.219	0.007	0.765
τ_WS_^**^ (4)	20 [20.56]	5.0 [5.0]	0.293	0.014	0.90
β_WS_ (4)	0.5 [0.515]	0.125 [0.125]	0.216	0.002	−0.20^†^
**AFFECTING MODEL INHIBITORY PATHWAYS**					
*h*_D_ (2)	6 [6.1155]	1 [1.0025]	0.217	0.005	−0.86
γ_S_ (3)	100 [102.309]	20 [20.050]	0.217	0.006	−0.86
*α*_Z_ (3)	0.05 [0.0514]	0.0125 [0.0125]	0.218	0.007	−0.80

Parameter symbols are as in Brown et al. (1999); SD = standard deviation. n/a not appropriate. Samples are of 50 simulated individuals. Population values for parameters are the values of the random normal variables specified in the simulating program. The same sample of standard random normal values was used in each simulation but was scaled to give the appropriate SD for the parameter, as reported above. Simulations for parameters marked with an

* used a different sample of random normal values; those for parameters marked

** were for a larger mean value, matching that used in the original paper; and those for parameters marked with a

‡ used a uniformly spaced set of parameter values, covering the specified range. Correlations marked with a

† *should be treated cautiously as clear, non-linearities were observed. Simulation output = change in normalized DA firing rate between UR and UNR trials, in DA spikes per 20 ms recording bin*.

Table 2 shows that for the simulation varying parameter *w*_PD_ the mean output was close to 0.2, thus reflecting an approximately 10 spike per second increase in DA firing on UR trials relative to UNR trials. The scale of this output variable is controlled largely by the neural spiking equation and associated parameters, which were taken (unchanged) from the published BBG model. The results for parameter *w_PD_* reveal that it satisfies the VSR constraint as it produces a correlation of 0.98 (0.95 in the second simulation, with a different random number seed) between the parameter and the DA spike rate change. This correlation was positive as predicted by the RST of extraversion, where extraverts have larger reactivity to reward signals, and the size of the excitatory response to incoming reward signals is captured by the parameter *w_PD_*. Furthermore, the simulation reveals a sizeable degree of variance in the simulated output: the *SD* was approximately 0.05 (0.04 in the alternative simulation), to accompany the mean value of 0.225 (0.214). We will take the fact that both our key constraints are satisfied as evidence that variation in *w*_PD_ (the excitatory drive from brain reward systems to midbrain dopaminergic cells) is a plausible candidate to be part of the psychobiological basis for extraversion. Moreover, variation in *w*_PD_ can therefore explain the observed (and replicated) correlation between extraversion and a difference in the FRN ERP waveform elicited by unpredicted reward and unpredicted non-reward trials in the Potts associative reward learning paradigm (Smillie et al., 2011; Cooper et al., 2014). Moreover, this same parametric variation might underlie the observed link between anticipatory pleasure and the FRN difference waveform (Cooper et al., 2014).

It is worth considering next the possible neurotransmitter system(s) which might mediate variations in values of the parameter *w*_PD_. The traditional view is that the excitatory inputs to midbrain dopamine neurons are glutamatergic while the inhibitory inputs are GABAergic (for up-to-date reviews see Watabe-Uchida et al., 2012; Covey et al., 2014). Such a view would suggest that the *w*_PD_ weight could reflect the efficiency/density of receptors at gluatamatergic synapses providing the excitatory drive to the cell bodies of the DA cells. However, there are nicotinic acetylcholine receptors on these DA cells as well, and they are activated by inputs from PPTN (Covey et al., 2014, Box 3), so the parameter might alternatively (or additionally) reflect the efficiency/density of nictonic receptors. More recent data suggest that there are also noradrenergic inputs to the DA cell bodies (see Covey et al., 2014, Figure 2B), but it remains unclear if they are activated by inputs on the excitatory reward input pathway of the BBG model. Indeed, the number of afferent regions regulating dopamine neurons appears to be much greater than previously thought (Watabe-Uchida et al., 2012).

The picture could be even more complex as, for example, there are pre-synaptic D1-type DAergic receptors on the terminals of the glutamatergic inputs to midbrain DA cells. When these receptors are stimulated (e.g., by ethanol at low concentrations) this increases glutamate release in the VTA, thus raising somatodendritic dopamine release, which further activates the pre-synaptic D1 receptors (Xiao et al., 2009). So, if these pre-synaptic DA neurons varied in their efficiency/density they are likely to vary in their responsiveness to naturally occurring somatodendritic DA release, and thereby contribute to glutatamatergic receptor effects on midbrain DA cells. Thus, it is possible that DA receptors could also be part of the basis for the variations in size of the DA cells' responsivity to incoming reward signals, that is summarized by parameter *w*_PD_ in the current model.

Next we will vary a series of other parameters in the model, one by one, and the findings from these simulations can be compared with the findings from the “benchmark” *w*_PD_ simulations above. While parameter *w*_PD_ is the final common path of the excitatory inputs to the DA cells, the BBG model (see Figure 3) has a number of excitatory input pathways, which each converge on this final pathway. We systematically added individual differences to each of these pathways in turn to explore their respective contributions.

There is a direct pathway from primary reward processing in the lateral hypothalamus (LH) to the PPTN (location 6) in the model. The relevant parameter in the model is *w*_*RP*_. This parameter controls the strength of the excitatory (primary reward) input from LH to PPTN; it can be thought of as reflecting an individual's sensitivity to unconditioned reward stimuli. The correlation between the *w*_*RP*_ parameter and the simulated output was 0.91, which satisfies the VSR. However, the amount of variance in the output parameter was much less than the simulation for *w*_PD_ (*SD* = 0.01, compared with 0.05 for *w*_PD_). However, the scale of the *w*_*RP*_ parameter used in the simulations was also about one fifth of that for *w*_RP_ (see Table 2), so it is unclear whether this parameter satisfies the MV constraint to the same extent as *w*_PD_. Moreover, the simulated relationship showed possible signs of non-linearities. To address these issues, we reran the *w*_RP_ simulation using a uniformly spaced set of parameter values, covering a much larger range of parameter values than were explored via the random normal variable (see Table 2). The results of this simulation revealed a clear linear relationship between the parameter and simulated output over the whole range of parameter values (*r* = 0.97) and an *SD* for the simulated output of 0.041. This level of simulated output variability is similar to that found for the simulations with parameter *w*_PD_. In this second simulation the *SD* for the *w*_RP_ parameter was (1.16), i.e., of a similar scale to that used in the simulations with parameter *w*_PD_ (see Table 2). Thus, we conclude that the *w*_RP_ parameter satisfies the VSR and MV constraints to a similar extent to parameter *w*_PD_.

In a similar fashion we also explored two other parts of the excitatory input pathways to the midbrain DA cells: locations 5 and 7 in Figure 3. In the case of location 5 this was the terminals of the indirect double inhibitory projections from VS to PPTN, represented in the model by the excitatory connection weight parameter, *w*_SP_^5^. In the case of location 7, this was the projection from LH to VS cells, represented in the model by the connection weight parameter, *w*_RS_. The findings are summarized in Table 2.

In the case of parameter *w*_SP_ the results show that this parameter satisfies the VSR (correlation = 0.98 c.f. 0.98; see Table 2) and MV (0.08 c.f. 0.05; see Table 2) criteria at least as strongly as did the simulations which varied parameter *w*_PD_. This occurred despite the fact that the simulations with *w*_SP_ added random normal variation to the parameter with a smaller *SD* (0.5) than was used in the simulations with *w*_PD_ (*SD* = 1.0). Moreover, the *w*_SP_ relationship with the simulated output remained strongly linear in a further simulation, which used a much wider range of parameter values for *w*_SP_ (see Table 2).

Intuitively, this result makes sense as the key outputs being simulated are DA cell firing changes resulting from conditioned reward stimuli conditioned at location 4 (Figure 3) and relayed to the PPTN via location 5. However, the cells on this pathway are thought to be GABAergic (Brown et al., 1999; Watabe-Uchida et al., 2012) with tonically active cells in the ventral pallidum (VP) inhibiting midbrain DA cell firing. These VP cells are thus inhibited by GABAergic outputs from the VS cells, thereby releasing the DA cells from their tonic pallidal inhibition. The model simplifies this indirect pathway via a single parameter (*w*_*SP*_) but if variations along this pathway were to underpin variation in extraversion and our endophenotype (the FRN difference wave), then this might imply variation in the effectiveness of the signaling of GABAergic neurons.

In the case of *w*_RS_ the simulation revealed a striking non-linearity in the relationship between the *w*_RS_ parameter and the simulated output. To explore this more fully, we repeated the simulations but using a set of values for *w*_RS_ which were uniformly spaced over a much wider parameter range (see Table 2). These showed that below a value of 0.8 there was a weak linear increase in the effect of the *w*_SP_ parameter on simulated output (which rose to a value of about 0.1). Then there was a large jump to simulated outputs around 0.22, which stayed flat over the rest of the range of values used for parameter *w*_RS_. This means that parameter *w*_RS_ failed the VSR criterion. If “naturally occurring” psychobiological parameter variations, underlying extraversion and equivalent to parameter *w*_RS_, were in the range that was functionally equivalent to *w*_RS_ values above 0.8, then this would mean that the parameter variation would have no effect on the endophenotype and a significant trait-endophenotype variation would be impossible to detect in real data. If the real psychobiological parameter values spanned either side of the 0.8 value (i.e., either side of the threshold which led to a step increase in the effect on simulated output), then one might expect a similar striking non-linearity in the relationship between the trait and the endophenotype. No such non-linearity was seen in the real trait-endophenotype relationships reported by our lab (Smillie et al., 2011; Cooper et al., 2014).

It is reasonably straightforward to work out why the effect of parameter *w*_RS_ on the simulated output should be non-linear. As explained in Supplementary Material, we made a few minor modifications to the original BBG model. One was to introduce a threshold in the activation of the VS cells before weight modification (at location 4, Figure 3) could occur; a threshold was not used in the original model and the weight modification was simply proportional to whatever activation of the VS cells was occurring. This change is more biologically realistic because, under three-factor learning rules of the kind used here, there should be post-synaptic depolarization to induce learning (Reynolds and Wickens, 2002); the threshold simply imposes this constraint. Therefore, for the other parameter values used in our simulations, when the value of parameter *w*_RS_ falls below a specific value (0.8, as noted above), there is not sufficient input to the VS cells for them to be activated enough to undergo weight change on their inputs from the cortical stimulus representations.

The above makes it clear that most of the pathways providing excitatory drive to the midbrain DA cells satisfy the VSR and MV constraints; thus there are several plausible candidates for the locus of the reported extraversion-related effects on the FRN. Sticking with the excitatory pathways, we next consider effects on parameters involved in actual weight modification, rather than the static weights we have considered to date. As already noted, our model uses a so-called three-factor learning rule for the weight changes at both location 4 and location 3 in Figure 3. One of those three factors is, of course, driven by the dopaminergic events set in train by a phasic change in firing rate of the midbrain DA cells. A logical proposal is that, at the locations in the model, the key events will involve post-synaptic DA receptor activation. Variation in the “trait” levels of such DA receptor activation has been proposed as a basis of extraversion (Depue and Fu, 2013). In the model used here the parameter *w*^*max*^_*S*_ can be seen as corresponding to the extent of dopamine receptor activation that can be induced by a fixed sized burst of increased firing (variable *N*^+^ in the model) from the midbrain DA cells (see BBG equation 2 and Supplementary Material for details). As shown in Table 2 above, parameter *w*^*max*^_*S*_ satisfied the VSR and MV criteria to the same extent as parameter *w*_PD_. The correlation between *w*^*max*^_*S*_ and the simulated output was strong (0.92) and variation in *w*^*max*^_*S*_ produced sizeable variation in the simulated output (*SD* = 0.042). This occurred despite the fact that the random normal variable used in the simulations for *w*^*max*^_*S*_ had a lower *SD* (= 0.25) than was used in the simulations varying *w*_PD_ (*SD* = 1.0). Therefore, these simulations confirm that post-synaptic DA receptor activation in VS is a plausible basis for extraversion-related effects on the FRN difference wave.

To conclude our exploration of the excitatory pathway effects we also considered other weight change parameters from BBG Equation 2; namely τ_WS_ and β_WS_. The parameter τ_WS_ is the overall rate constant for the weight changes in Equation 2. However, Table 2 reveals that the simulations varying this parameter produced a more modest positive correlation with simulated output (0.765) but crucially a weak level of simulated output variance (*SD* = 0.007; c.f. 0.052 for parameter *w*_PD_). However, the amount of parameter variance in this simulation was less (*SD* = 0.25) than that used in the simulations with parameter *w*_PD_ (*SD* = 1). Therefore, we ran a further simulation with parameter τ_WS_ and dramatically increased both the mean (from 1.0 to 20.0) and *SD* used (from 0.25 to 5). This produced a modest doubling of the *SD* of the simulated output (from 0.007 to 0.014) which still compared unfavorably to the variance created by varying parameter *w*_PD_. We therefore conclude that the overall rate constant of the weight change equation at VS neurons fails the MV criterion.

Parameter β_WS_, by contrast, controls the rate of weight decrease when a phasic decrease in DA cell firing occurs (given by variable *N*^−^ in the model). This could also be seen as an effect mediated by DA receptors in the VS (Bromberg-Martin et al., 2010). However, Table 2 shows that simulations varying this parameter fail the VSR constraint (the parameter-output correlation was −0.20, with signs of non-linearity). Moreover, varying this parameter induced very little variance in the simulated output (*SD* = 0.002) so the MV criterion was also failed.

Next we consider simulations on the inhibitory pathways of the model. We start by considering an effect at location 2 in Figure 3. At this location the timed reward prediction, computed by the striatal striosomal spectral timing array, acts to inhibit the firing of the midbrain DA cells at a time, following specific stimulus events, when a rewarding event (one that increases DA cell firing) has occurred in the past. The inhibitory effect of the striosomal output signal is directly scaled by parameter *h*_D_ in BBG equation 6. This parameter can be seen as reflecting the effectiveness of the inhibition; and as the pathway from the striosomes to the DA cells is GABAergic (Brown et al., 1999; Watabe-Uchida et al., 2012) one could view it as reflecting the effectiveness and/or density of GABAergic receptors on the DA cells.

The larger the value of parameter *h*_D_ the more inhibition of DA cells there is; thus the correlation with the simulated output (increases in simulated DA firing on UR trials minus the decrease in simulated DA firing on UNR trials) is expected to be negative. If *h*_D_ were to be a candidate parameter related to the trait of extraversion, then a high value of *h*_D_ would have to be related to low levels of extraversion. In this way, *h*_D_ variation could explain the positive correlation, in real data, between extraversion and the FRN difference wave.

Table 2 reveals the results of our simulations varying *h*_D_. The correlation between *h*_D_ and the simulated output was indeed negative, as predicted (*r* = −0.86). However, varying the parameter had very little effect on simulated output (*SD* = 0.005), about a tenth the *SD* for simulations varying parameter *w*_*PD*_ (*SD* = 0.052). This occurred despite the fact that the amount of parameter variance used was the same in each case (the *SD* used for varying both *h*_D_ and *w*_PD_ was 1.0). We therefore conclude that variations in the effectiveness of striatal inhibition of DA cell firing fails the MV criterion, and such variations are thus not a plausible candidate for the cause of extraversion-related effects on the FRN difference wave.

We can, as a minimum, make this statement about the plausibility of *h*_D_ relative to many of the parameters, analyzed earlier, in the model's excitatory pathways. It is perhaps not surprising that these variations in the inhibitory pathway are less effective than variations at points in the excitatory pathway, in terms of causing variations in simulated DA firing. This follows because midbrain DA cells have a low spontaneous rate of firing and so the scope for reduced firing is less than that for increased firing: the dynamic range is less in the inhibitory direction than in the excitatory direction (Glimcher, 2011).

Next we consider parameters affecting the learning (i.e., weight change) in the inhibitory pathway of the model. This weight change occurs in the glutamatergic pathways carrying inputs from cortical cells, activated by the stimuli. In Figure 3, this learning takes place at location 3. The weight learning is governed by equation 6 in the BBG model. A key parameter in this equation is the striosomal learning gain, γ_S_. This parameter scales the amount of weight change produced at striosomal receptor sites that are eligible to undergo learning (i.e., at sites that satisfy the pre-synaptic and post-synaptic activation criteria). The third factor which is needed for weight change is dopaminergic activity triggered when a burst of increased DA cell firing activates receptors at the striosomal terminals (such a burst is denoted by variable *N*^+^ in the model). The parameter γ_S_ thus can be seen as reflecting the degree of post-synaptic DAergic receptor activation in the striosomes. In our simulations variation of γ_S_ is expected to be negatively related to our simulated output variable (for similar reasons to those give earlier for simulations varying parameter *h*_D_).

Table 2 gives the result of the simulation varying γ_S_. As expected the correlation with simulated output was negative (−0.86), but there was very little resulting variation in the simulated output (*SD* = 0.006). This was about a tenth of the variation produced by variations in the parameter *w*_PD_, for example; and this difference occurred despite much more variance being added to parameter γ_S_ in this simulation (parameter *SD* = 20) than was added in the simulation which varied *w*_PD_ (parameter *SD* = 1.0). As with parameter *h*_D_ in the inhibitory pathway, we conclude that parameter γ_S_ fails the MV criterion, at least relative to the variance created by varying numerous parameters in the excitatory pathways of the model. As we have interpreted parameter γ_S_ as reflecting the extent of post-synaptic DA receptor activation in the striosomes, we tentatively conclude that variation such post-synaptic receptor activation is not a plausible basis for extraversion-related effects on the FRN difference wave recorded from EEG. This could be an important qualification to one of the earlier conclusions: namely that post-synaptic DA receptor activation in the VS was a plausible basis for these effects. If extraversion is related to DA receptor activation in some striatal areas, but not others, then this is a worthy target for future studies.

Our final simulation in the inhibitory pathway varied the parameter *α*_Z_. This parameter is the weight change rate parameter within BBG equation 6; this controls the overall rate of weight changes in the weights at the terminals of the stimulus afferents to the striosomal sites (i.e., at location 3). In the same way as we observed earlier for weight change rate parameter β_WS_ on the excitatory pathway, variations in *α*_Z_ failed the MV criterion (see Table 2 for details). Perhaps weight change rate parameters are generally ineffective at inducing variation in simulated outputs (at least in three-factor learning equations). The ineffectiveness of a learning rate parameter, as a candidate for individual differences, was what we observed in our first foray in to DCN modeling over a decade ago (Pickering and Gray, 2001).

## General discussion

### Limitations of the current work and next steps

The current work is the first detailed attempt to tackle the difficult problem of exploring individual differences using the DCN framework in an indirect way. The approach is limited by the fact that, in using a biologically anchored computational model, the model necessarily has a large number of parameters (see Supplementary Material). This means that we cannot fit the model to the performance of individual participants across the trials of the experiment. If we could do so, then we could explore which fitted model parameter correlated best with variation in our phenotypic trait of interest (here extraversion), measured in the same participants. This “direct” approach is tractable only with simple models with few parameters (see Brazil et al., 2013, for an example of using the direct approach in relation to psychopathic personality traits).

The indirect approach, adopted here, tries to uncover model-based constraints on which psychobiological features are more vs. less plausible causes of variation in the endophenotype. We concluded above that parameter variation, along most of the pathways providing excitatory drive to midbrain DA cells, is more likely to create detectable variance in our (simulated) endophenotype than would variation along the pathways acting to inhibit midbrain DA cell firing. However, even this reasonably broad conclusion, would be called into question if the underlying model was wrong in a major way; the detail and specificity of the model used mean that it is almost certain to be wrong and/or incomplete in several details. To address this we might use a variety of related models, with some shared features to the BBG model, each of which is capable of simulating the endophenotype studied here. If several of these alternative models showed the same broad conclusions with respect to individual differences, then this would imply that the findings are not tied too closely to the specifics of the BBG model. To do this would be a major undertaking. Therefore, as an alternative next step, we have begun an attempt to boil down the BBG model to a minimal set of components, with the smallest number of parameters possible. We will then be able to see if the same conclusions are obtained with our “reduced BBG” model. This work is currently incomplete; but early results suggest that our conclusions about the effectiveness of excitatory vs. inhibitory pathway variance holds up. Assuming these preliminary findings with the reduced model are confirmed, next we will attempt to use our reduced model in a direct fashion: fitting the model to individual participant FRN data trial by trial, obtaining a best-fitting set of parameters for each participant. Then we will be able to see which parameter explains the most variance in the extraversion trait scores of the participants.

There are other possible limitations, even assuming the BBG model is correct in its basic assumptions. We have been comparing the effects of adding variance to parameters when these parameters were sometimes on differing scales. Because all the parameters took positive values, once we had established a good set of mean parameters (by trial and error), we were limited in how much variance we could add to certain parameters. For example, if a parameter (such as *w*_SP_) had a mean value of 2.0, we added variance using a random normal variable with an *SD* of 0.5 (= 2.0/4), so that no simulated individual would be likely to have negative parameter values. Another parameter (such as γ_S_) had a mean value of 100; the random normal variable we used could have a much larger variance (we used an *SD* of 20), while still ensuring that this was never likely to lead to a value of γ_S_ which fell below 0. While we believe that our conclusions about the effects of parameter variation are sound, despite these scaling issues (see Table 2), this is a potential concern. One possible solution would be to try tweak the model so that all parameters were on the same scale, thus allowing the same amount of variance to be added to each parameter. Based on our trial and error attempts which led to the mean parameter values adopted here, we do not think that such an approach would be achievable.

Our arguments about the constraints operating on our models were based on a central psychometric assumption: namely, that a single parameter might underlie variation in aspects of measured extraversion and also contribute substantial variance to our endophenotype. An alternative account would be to argue that multiple independent parameters might each contribute additively to variance in extraversion, while also contributing additively to variance in our endophenotype. This certainly should make it easier to observe correlations between traits and endophenotypes in the lab. It is unclear to us how much this would “release” our modeling from the VSR constraint, given that we were able to generate a typical correlation of 0.3 between a trait and an endophenotype only by making very generous (and in our view unrealistic) assumptions about the number of psychobiological features contributing variance to the trait and endophenotype (see our earlier example). Allowing more than one model parameter to contribute to trait-endophenotype covariance might simply serve to offset the overly generous assumptions we made in the first place. However, simulations using the model developed in this paper, which allowed more than one parameter to vary, showed that several combinations of parameters did not produce additive effects on simulated output variance (details on request from first author).

The VSR constraint outlined in this paper was rather vague: we did not attempt to place a firm cut-off for the required strength of the relationship between the manipulated parameter and the simulated process. There is simply not enough information about the number of underlying causes affecting extraversion and any endophenotype for us to do this with confidence. We illustrated the VSR via some psychometric calculations in the paper, based on very generous assumptions. Under these assumptions, with a perfect relationship (*r* = 1.0) between the simulated parameter and the simulated process, the correlation between the endophenotype and Extraversion would be at most 0.3. This would drop to 0.15 if the correlation between parameter and process were only 0.5. Given the generosity of the assumptions behind these calculations we expect that the relationship would have to be near perfect (very close to 1.0) in order to be able to detect a reliable trait-endophenotype correlation (at least with typical sample sizes used in real studies). Indeed, for some of our candidate parameters the relationship was 0.95 or greater (see Table 2). We did not rule out other parameters entirely on VSR grounds, even though the relationship was numerically somewhat weaker (e.g., 0.86–0.92). In our view attempting to adjudicate between parameter-process correlations in this range of values is not likely to be especially fruitful. We would prefer to test between these candidate parameters by following step 5 of the DCN framework (as sketched briefly below).

Another limitation might be that the BBG model produced largely linear relationships between the varying parameter values and the simulated levels of the endophenotype. There is considerable evidence that dopaminergic neurotransmission may exert non-linear effects on a variety of phenomena (e.g., see Durstewitz and Seamans, 2008). This could be a major issue for the current model if extraversion and the FRN were demonstrated to have a non-linear relationship, perhaps because of the influence of some dopaminergic process. The E-FRN relationships in our lab (reported above) have been linear in nature, although some non-significant relationships between extraversion(-like) traits and FRN(-like) phenomena have been reported in earlier papers, as discussed in the introduction.

In one recent paper, Mueller et al. (2014) reported extraversion and dopaminergic drug effects on the FRN, which was elicited during a complex, multi-phase, computerized ball-catching game. In one phase of their task, the FRN measure showed a marginally significant (*p* = 0.06) positive association with extraversion (specifically a measure of *agentic* extraversion) amongst participants given a placebo. However, amongst participants given a low dose of sulpiride (a selective D2-receptor antagonist), the FRN extraversion relationship was negative and non-significant (but significantly different from that observed in the placebo condition). While this study gives support to the view that dopaminergic processes are involved in the correlation between extraversion and the FRN, the authors view it as consistent with a curvilinear relationship between dopaminergic processes and phenomena like the FRN. The BBG model used in the present study would need some modification to capture this non-linearity. A future study using dopaminergic drug (such as sulpiride) and the Potts et al FRN task could confirm whether the Extraversion and FRN relationship we have reported is abolished under D2-receptor blockade. If this was confirmed then attempts to model this drug effect could be attempted.

More generally, when applying the DCN approach in other specific situations, it might be found that the relationship between parameter variation and the simulated endophenotypic process was non-linear. In that case alternative methods for assessing the relationship would be needed, as the use of linear correlational methods, as deployed here, would be inappropriate. Methods such as mutual information (Cover and Thomas, 1991) might be much more useful in these contexts.

Notwithstanding the difficulties and limitations just noted, having an explicit model makes future studies using variants of the Potts associative reward learning task potentially more powerful. For example, one could carry out psychopharmacological studies using this task with human participants and predict the effects of drugs (and their moderation by extraversion) more precisely. However, a more immediate prediction is available from inspection of Figure 5, which reveals that the model predicts that DA firing increases are prominent, during S1, for the PR and UNR conditions, while very little change in DA firing during S1 is predicted for the UR and PNR conditions. It should be possible to assess the FRN during S1 during the Potts associative reward task. It would then be straightforward to construct an S1 FRN difference wave (from PR and UNR trials minus UR and PNR trials). This FRN difference wave could then be correlated with extraversion scores. We are currently undertaking an analysis of such an S1 FRN difference wave in our lab. Before doing these analyses, the model can make predictions for each candidate individual differences parameter; this prediction will concern the strength of the correlation between the parameter and simulated DA cell firing rate changes during S1. The key question is how this correlation compares with the corresponding correlation between the parameter and the simulated S2 DA cell firing rate change.

For example, from the current simulation data, we found for parameter *w*_PD_ that the relationships with the S1 output were even stronger than those with the S2 output reported above. Using the same sample of random values for the parameter, the mean value of the DA cell firing change was greater (0.704 c.f. 0.225) the correlation was even closer to 1 (0.9998 c.f. 0.98) but more importantly the *SD* of the simulated output was strongly increased (0.139 c.f. 0.052). Thus, if the psychobiological feature corresponding to parameter *w*_PD_ underpins the observed correlation between extraversion and the FRN difference wave recorded at S2, then the correlation between extraversion and the FRN difference wave recorded at S1 should be stronger. It is possible to make these systematic comparisons for all the candidate individual differences parameters reported above; this illustrates the power of using a formal computational modeling framework.

One further remark seems especially noteworthy: the modeling presented in this paper is an attempt to understand trait correlations with an FRN difference wave. This ERP phenomenon was chosen as a modeling target on the basis of the widespread belief that the FRN is modulated by the phasic activity of midbrain dopaminergic neurons, and there are well-established models of dopamine neuron firing (such as the BBG model used here). One might simplistically conclude that the FRN difference wave is a “dopaminergic phenomenon”; by extension the observed correlation between the difference wave and extraversion might be used to argue that extraversion has a dopaminergic basis. However, the use of the model showed that extraversion-related variation in our FRN difference wave might derive from variation in the action of many other neurotransmitters, via their receptor sites on the DA cells themselves, or at points on the afferent pathways acting upon them. Above, we have made a case that the trait effects could lie in any of the following: glutamatergic, cholinergic, GABAergic, as well as dopaminergic, processes. This valuable general insight flows directly from having an explicit and biologically-anchored computational model. It is hoped that the prospect of more nuanced explanations, of this kind, will persuade more researchers to use similar computational approaches. We are convinced that they are one route to gaining more sophisticated insights into the psychobiological underpinnings of personality traits.

It should also be pointed out again that we have not simulated the mechanism by which midbrain DA firing rate changes actually modulate the FRN (generated in the ACC). We stressed earlier that this modulation has to be via a non-dopaminergic mechanism in order to occur quickly enough. It is therefore, of course, possible that the parameters controlling this unmodeled step (perhaps involving co-release of glutamate by these DA cells) might be ones which explain the relationship with extraversion. If this were correct, then this would be another illustration of how extraversion might be in part dependent on non-dopaminergic processes of neural transmission.

Finally, we close the discussion by considering how we might pursue steps 5 and 6 of the DCN using variations of the current model.

### Step 5: test the most plausible, specific model parameters for their ability to simulate variation in other distinct, but conceptually related, endophenotypes

As already noted, the current research emerged from the backdrop of theorizing which linked extraversion in some (often underspecified) way to “reward-based learning.” An obvious next step would be to see which of the candidate parameters, reported here, might also have the potential to underlie the associations between extraversion and performance on reward-dependent learning tasks. To do this, we have begun to modify the BBG model so that it is able to simulate tasks requiring reward-guided choice responses by the participant. To do this, another ascending projection from the midbrain DA cells could be used to control the learning of stimulus-response associations, taking place in another “response selection” module of the model. The learning rule used, again based on phasic firing changes of the midbrain DA cells, could be the same as that used in the excitatory pathway of the BBG model (i.e., the one operating at location 4 in Figure 3). We have unpublished data from a probabilistic reward-learning task in which extraversion is positively associated with reward-guided learning performance, and on another task (where the contingencies change without warning during the task) in which extraversion has a significant negative effect on task performance. Being able to simulate both these effects, and the FRN, via the same parameter, will be a significant challenge, but we expect that it will reduce the number of candidate parameters dramatically.

### Step 6: explore whether, and in what contexts, the proposed parameter variation can simulate aspects of extraverted behavior in “toy” models

The psychometric model of extraversion that we have advocated (see Figure 1) makes it clear that we are *not* attempting to propose candidate psychobiological parameters which could account for all the reliable measured variation in extraversion. Rather, we have proposed a method for unearthing psychobiological parameters that might possibly underpin variance in responding to a small subset of extraversion items. In this light, it seems possible to us that the parameters we have concentrated on (i.e., ones that influence reward prediction learning) might explain part of the reason why extraverts learn to make more sociable responses than do introverts when meeting people.

To test this idea, we are currently using a simplified, response-dependent variant of the BBG model and using it to simulate “toy” social interactions between a “modeled individual” who “meets” a number of other simulated individuals. The modeled individual in these simulations can respond to the people they meet using a small repertoire of possible responses (e.g., either with a sociable, neutral or “shy” response, each of which might be equiprobable at the outset of the simulation). We make the reasonable assumption that any sociable responses the modeled person makes are more strongly socially reinforced than their non-sociable responses. In the simulations, the modeled person meets a computer-controlled set of “individuals” who provide social reinforcement, after the modeled person has made their initial response. The individuals who are being met are programmed to differ systematically in the mean and variance of the social reinforcement they typically provide (over repeated encounters).

We are varying the nature of the modeled individual by adding variance to some of the candidate parameters that were reported in the current paper. Thereby, we can make predictions about which types of individuals will elicit the greatest, and least, degree of sociable responding from extraverts, and how introverts will differ in their responses to these same simulated people. This work is ongoing but the predictions which will emerge can then be tested in a lab experiment in which the participants would take the role of the modeled individual. The computer-controlled people that they “meet” would be the same as those used in the simulations and the same range of responses would be available. In this way we may be able to show that our candidate parameters are likely to affect (parts of) the phenotype, as well as affecting the endophenotypes we have used as proxies for that phenotype.

## Author note

The ideas and modeling approach behind this work were initially developed while Francesca Pesola was a doctoral student under the supervision of Alan D. Pickering. The specific application and models presented in the current paper were developed by Alan D. Pickering.

### Conflict of interest statement

The authors declare that the research was conducted in the absence of any commercial or financial relationships that could be construed as a potential conflict of interest.

## Footnotes

^1^Further power enhancement would occur if the endophenotype was also measured more reliably than the phenotype.

^2^There are number of similar event related potentials that have been called a variety of different names: medial frontal negativity; frontal negativity; error-related negativity etc. For a comparison between these potentials see Cavanagh and Frank (2014), who note their “overwhelming similarities.” In this paper we focus on the FRN.

^3^Technically, we focus on the so-called model free RL models (Skatova et al., 2013) as the direct link between DA firing and RPE signals has usually been made for that class of RL models.

^4^As noted above, the FRN (and related waveforms) have widely been argued to arise from generators in structures such as the anterior cingulate cortex (ACC; see Holroyd and Coles, 2002) that are densely innervated by ascending projections from midbrain DA cells. Also as discussed earlier, we assume that DA cell firing changes modulate the FRN in the ACC but, in order to do so with the required rapid temporal dynamics, this modulation occurs via non-dopaminergic mechanisms (see also Ullsperger et al., 2014).

^5^Note that represent this pathway via a single excitatory connection is a simplification of the inhibition of inhibition that is actually occurring. This point is discussed later in the text.
