# Supplementary material for: Modeling dopaminergic and other processes involved in learning from reward prediction error: contributions from an individual differences perspective
Source: Front Hum Neurosci. 2014 Sep 30;8:740. doi: 10.3389/fnhum.2014.00740 (PMC4179695; doi:10.3389/fnhum.2014.00740)
Supplement: Supplementary file 1 [file Presentation1.PDF]

Pickering & Pesola: Supplementary Material

The full Matlab code which runs the model described in this paper is available from the first author on request. The model performed a series of simulated learning trials. The differential equations of the BBG model were simulated using a first order Euler method with a timestep of 1 msec. Each simulated trial lasted 3000 msec. Stimulus S1 onset at 300 msec into the trial and was active for 500 msec; stimulus S2 onset at 1100 msec into the trial and was active for 500 msec; and the reward onset at 1900 msec and was active for 500 msec. These signals were square waves which, in the case of S1 and S2, triggered simulated neural activation as described by equations 1 and 2 in the main text of the current paper. The reward signal was also a square wave with a value of 1.5 when the reward occurred and 0 otherwise; in the original model the reward signal took a value of 1.0.

We will go through the equations given in (Brown et al., 1999), in numbered order, noting the minor changes we made. In BBG equation 1, the value of 1 (which specifies the upper value that can be reached by a ventral striatal neuron) was replaced by 1.5 in the current simulations. There are 2 cortical input neurons in the current simulations[1], one for each stimulus, which both project to a single ventral striatal (VS) neuron. Equation 2 of the BBG model controls the learning of the modifiable synaptic weights between the cortical and VS neuron (at location 4 in Figure 3 in the main text). In the original BBG equation 2, the learning took place when the VS cell was activated above zero. In our simulations we introduced a threshold so that learning occurred only when the VS cell was activated above this threshold (0.95).

Equation 6 in the BBG model describes the activation of the single midbrain DA cell, denoted *D*. In the original model this equation was such that the value of *D* was a non-linear, saturated function with a maximal upper value. This is achieved because excitatory inputs driving changes in activation are scaled by a term (1-D), which means that as *D* approaches 1 a given excitatory input produces less and less change in activation. Similarly, suppression below the baseline tonic firing rate by inhibitory inputs was also squashed in a similar way by being multiplied by a term ($h_D + D$). As we were exploring the variation of *D* as the critical output of our model, we wanted to maximise the range of values it could take and maximise its responsiveness to excitatory and inhibitory inputs. Therefore, we removed the non-linearities in Equation 6 in our simulations, replacing (1-*D*) by 1, and

[1] As is typical in computational neuroscience, we model small numbers of neurons. The activations and outputs of the modelled neurons are not taken literally to be single cells, but are more realistically viewed as representing the mean activity of a population of neurons of that type.

($h_D + D$) by h_D. In Equation 11 of the BBG model specifies the activation of the spectral timing mechanisms at sites on the striosomal cells. In the original model any output from the stimulus cells activates the spectral timing. In the main text, we described how we controlled the activation of the spectral timing such that only when the outputs from the stimulus cells exceeded a threshold value was the spectral timing activated. For the parameters used in our simulations (see below), we used 22 sites on a striosomal cell in order for there to be adequate temporal precision in the spectral timing, and an ability to span the time intervals occurring in the simulated task (n in BBG Equation 11 was thus 22 in our simulations). All the other equations we used were the same as those specified in the original BBG model.

Basic Model Parameters

In the table below the values for the model parameters are given. The values in the table were those adopted for the basic simulations. If the value we used was changed from that given in Brown et al. (1999), then the original value is shown in parentheses. The changes were made so that stable simulations of the current task were possible, given the minor modifications to the equations described above. For the individual differences simulations certain key parameters were given values that were (usually) distributed like a normal random variable, with the value in the table being the mean, and using a standard deviation (s.d.) specified as in Table 2 in the main paper.

**Symbol**	**Description**	**Value**
α_r	Striosomal spectrum spacing	25.0 (50.0)
β_r	Striosomal spectrum offset	1.0
Γ_G	Calcium spike threshold	0.495 (0.37)
α_G	Calcium activation rate	5.0
β_G	Calcium passive decay rate	20.0
B_G	Calcium concentration maximum	5.0
α_y	Calcium recovery rate	1.0
β_y	Activity-dependent calcium inactivation rate	80.0
Γ_y	Calcium inactivation threshold	0.16 (0.18)
Γ_S	Striosomal output threshold	0.2
γ_S	Striosomal learning gain	100 (10000)
α_z	Striosomal learning rate	0.05 (0.1)

w_{RS}	Hypothalamus-to-Ventral Striatum synaptic weight	1.5 (1.2)
τ_S	Ventral striatal cell response time constant	30.0
τ_{WS}	CS-to-ventral striatal learning rate	1.0 (20.0)
WS_{max}	Maximum CS-to-ventral striatal synaptic weight	1.0 (2.5)
β_{WS}	CS-to-ventral striatal weight decay rate	0.5 (0.2)
A_S	Ventral striatal activity passive decay rate	0.7
Γ_N	Phasic dopamine signal threshold	0.0
τ_P	PPTN cell response time constant	20.0 (200.0)
τ_{UP}	PPTN afterhyperpolarization time constant	4.0
τ_D	Dopamine cell response time constant	15.0
w_{PD}	PPTN-to-Dopamine cell synaptic weight	5.0 (50.0)
w_{SP}	Ventral striatal-to-PPTN cell synaptic weight	2.0
w_{RP}	Hypothalamus-to-PPTN cell synaptic weight	0.8
w_{UP}	PPTN afterhyperpolarization gain	50.0 (140.0)
Γ_P	PPTN output signal threshold	0.135
$\tau_{\bar{D}}$	Baseline average dopamine time constant	4.0
I_D	Tonic input to dopamine cell	0.15
h_D	Dopamine cell maximum hyperpolarization	6.0 (0.1)
V_I	Integrate-and-Fire (IAF) model output	0.5
C	IAF model membrane capacitance	0.025
σ_{noise}	IAF Gaussian noise input	0.4
R_{DA}	IAF dopamine cell membrane resistance	80
